# YTHDF1 promotes p53 translation and induces ferroptosis during acute cerebral ischemia/reperfusion through m^6^A-dependent binding

**DOI:** 10.1007/s10565-025-10061-3

**Published:** 2025-07-01

**Authors:** Xinyu Chang, Bingwu Li, Wanxu Huang, Aixia Chen, Shengmin Zhu, Yueyang Liu, Xiaoling Liu, Jingyu Yang, Dan Ohtan Wang

**Affiliations:** 1 Wuya College of Innovation, Shenyang Pharmaceutical University, Shenyang, PR China; 2 Department of Pharmacology, Shenyang Pharmaceutical University, Shenyang, PR China; 3 Biology Program, Science Division, New York University Abu Dhabi, Abu Dhabi, United Arab Emirates

**Keywords:** I/R injury, m^6^A, YTHDF1, p53, Translation, Ferroptosis

## Abstract

The rapid escalation of oxidative and nitrosative stress during ischemia/reperfusion (I/R) triggers neuronal damage, leading to severe neurological deficits and long-term disability. N6-methyladenosine (m^6^A), a highly abundant RNA modification in the brain, undergoes dynamic changes following acute I/R injury, and regulates stroke pathogenesis and neurological outcomes. However, the molecular mechanisms by which m^6^A influences acute I/R injury responses remain elusive. Our study reveals that the expression of key I/R pathogenesis pathways positively correlates with the expression of m^6^A reader proteins. Modulating expression of YTHDF1, a neuron-enriched reader protein of m^6^A, results in bidirectional changes in oxidative stress response and neuronal viability under I/R conditions. We have identified *p53* mRNA as a critical target of m^6^A methylation and YTHDF1, driving the translation of p53 protein in a context- and m^6^A-dependent manner, which exacerbates oxidative stress and ferroptosis. This novel mechanism suggests the potential of targeting the m^6^A reader protein as a strategic avenue for developing neuroprotective therapies to mitigate I/R injury.

**Graphical abstract:**

m^6^A-dependent YTHDF1 binding to *p53* mRNA promotes its translation and ferroptosis during acute cerebral ischemia/reperfusion (I/R)

Critical points:

• I/R upregulates YTHDF1 expression and its binding to m^6^A-modfied *p53* mRNA;

• Binding by YTHDF1 promotes translation of *p53* mRNA and induces ferroptosis;

• AAV-mediated knockdown of YTHDF1 alleviates I/R-induced neuronal damage in acute phase.

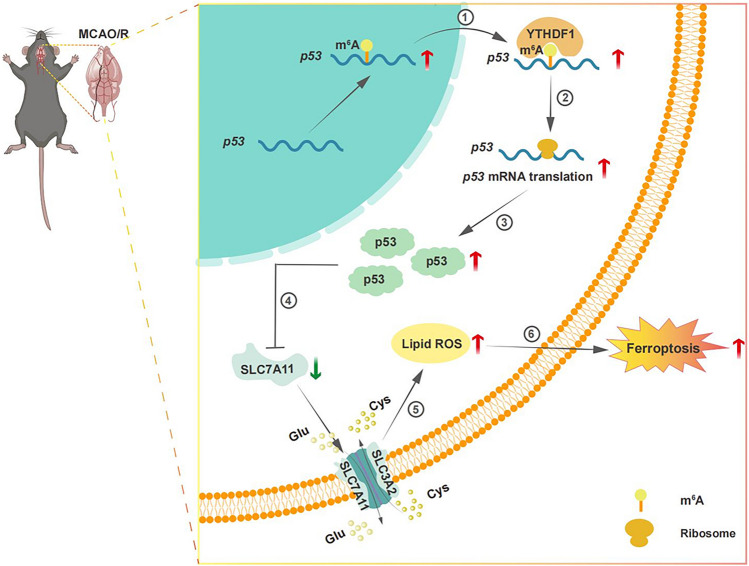

**Supplementary Information:**

The online version contains supplementary material available at 10.1007/s10565-025-10061-3.

## Introduction

Stroke is a major cause of mortality and disability globally, impacting millions of patients annually. The majority of incidents can be classified as ischemic strokes, which occur due to an acute blockage of blood flow in the brain (Collaborators 2021). Early revascularization using intravenous thrombolysis (IVT) or endovascular thrombectomy (EVT) is the primary medical intervention for treating acute ischemic stroke (Mosconi and Paciaroni 2022). However, beneficial outcomes are significantly limited by the narrow treatment window and the risk of deleterious reperfusion injury (Group et al. 2012; Zhou et al. 2023). Neuroprotective agents designed to inhibit ischemia/reperfusion (I/R)-induced pathophysiological cascades are critical, but few have been verified to improve neurological outcomes in clinical trials (Zhou et al. 2018). The high failure rates highlight the complicated etiology and pathophysiology of I/R, and calls for new therapeutic approaches (Lin et al. 2016; Zhou et al. 2018).

In the last decade, studies on post-transcriptional regulation by microRNAs and long non-coding RNAs have opened new possibilities for targeting gene network regulation in stroke (Bao et al. 2018; Mirzaei et al. 2018). RNA pathways with multi-target capacities within heterologous cell populations may offer more effective therapeutic intervention strategies. N6-methyladenosine (m^6^A), an abundant messenger RNA modification, marks thousands of RNAs in the mammalian brain and regulates their stability and translation (Livneh et al. 2020). The pleiotropic roles of m^6^A modification in multiple cellular injury signaling cascades have been reported, including DNA damage, oxidative stress, mitochondrial dysfunction, and cell death, offering insights into the mechanisms of its therapeutic potential in treating strokes and traumatic brain injury (Liu et al. 2022; Perlegos et al. 2022; Xiang et al. 2017; Yu et al. 2020; Zhang et al. 2021). In 2019, Chokkalla et al. demonstrated that m^6^A increases in the penumbra following transient focal ischemia, altering the methylation status of hundreds of mRNAs involved in inflammation, apoptosis, and transcriptional regulation (Chokkalla et al. 2019). In the following years, several labs reported collectively more than one thousand differentially methylated coding and noncoding RNAs that are functionally enriched in TLR, PPAR, and MAPK signaling pathways and inflammation (Shao et al. 2022; Yi et al. 2021). A recent ischemic stroke genome-wide association study has identified m^6^A-single-nucleotide polymorphism (m^6^A-SNP) candidates that are linked to I/R-associated risk (Zhu et al. 2021). These studies support the involvement of` m^6^A RNA modification in the etiology and pathophysiology of I/R.

m^6^A dynamics is regulated by specific writer (methyltransferases), eraser (demethylases), and reader proteins (Yang et al. 2018). Si et al. have shown a transient elevation of METTL3 (writer)-mediated m^6^A that facilitates stress granule formation and enhances cell survival during the early stages of ischemic stroke (Si et al. 2020). The demethylase FTO (eraser) decreases after I/R, potentially contributing to the global increase in m^6^A levels (Yi et al. 2021). Downregulation of FTO expression promotes the apoptosis of OGD/R neurons while upregulation mitigates neuronal damage via MEG3 expression and the NLRP3/caspase-1/GSDMD signaling pathway of neuronal pyroptosis (Xu et al. 2020a; Yan et al. 2024). The m^6^A eraser protein ALKBH5 also suppresses neuronal damage. Knocking down ALKBH5 in neurons led to the hypermethylation of *Bcl2* anti-apoptotic factor mRNA and its degradation, aggravating neuronal damage (Xu et al. 2020a). ALKBH5 deficiency also exacerbates ER stress, neuroinflammation, and apoptosis through modulating the STAT5/PERK/EIF2alpha/CHOP signaling pathway (Liu et al. 2024). Zhang et al. showed that overexpressing the nuclear m^6^A reader YTHDC1 mitigates I/R by destabilizing PTEN, thereby relieving its suppression of anti-apoptosis factors (Zhang et al. 2020). The YTHDF family of m^6^A readers comprising YTHDF1, YTHDF2, and YTHDF3, has also been associated with I/R injury (Li et al. 2022; Pang et al. 2023; Shen and Yue 2024; Zheng et al. 2020). These studies suggest that cellular injury could be mitigated by targeting proteins involved in m^6^A signaling pathways. However, to fully explore the therapeutic potential of m^6^A, an advanced understanding of its regulation and connection to downstream effectors of I/R-induced neuronal damage is necessary.

This study aims to elucidate the relationship between I/R-associated cell death pathways and dynamic signaling through m^6^A. Using in vivo and in vitro models of I/R, namely middle cerebral artery occlusion-reperfusion (MCAO/R) and oxygen–glucose deprivation-reperfusion (OGD/R), we identified m^6^A- and YTHDF1-dependent upregulation of p53 within 24 h following I/R. The increased YTHDF1 expression and hypermethylation of *p53* mRNA after OGD/R strengthened the binding between the target mRNA and m^6^A reader protein by 34-fold. Notably, I/R-triggered p53 expression, oxidative stress, ferroptosis, and infarct volume were reduced following AAV-mediated knockdown of neuronal YTHDF1.

## Materials and methods

### Animals and MCAO/R

All animal procedures were performed strictly in accordance with the Institute for Experimental Animals at Shenyang Pharmaceutical University. Before surgery, 6–8 weeks old mice were randomly divided into 4 groups (*n* = 6/group). With the exception of the Sham group, the remaining 3 groups were subjected to MCAO/R surgery and were divided into Re-0 h, Re-6 h and Re-24 h groups according to different reperfusion time points. The rearing environment and MCAO/R surgical procedures employed are detailed in the supplementary information.

### Methylated RNA immunoprecipitation sequencing (MeRIP-seq)

Total RNA was isolated from cerebral cortical peri-infarct tissue of sham, MCAO/Re-0 h, MCAO/Re-6 h and MCAO/Re-24 h mice with Trizol reagent (TaKaRa, Japan) according to the manufacturer's instructions. 10 μg total RNA from each sample was DNase I treated and fragmented to approximately 100 nt length using ZnCl_2_ solution (Invitrogen, USA). 1/10 of the fragmented RNA was used as INPUT (input RNA) and the remaining was immunoprecipitated with 3.5 µg of m^6^A antibody (Millipore, USA) as IP (immunoprecipitated RNA). cDNA libraries were prepared using the SMARTer Pico PCR cDNA Synthesis Kit (Clontech, USA) according to the manufacturer's protocol, and sequenced using Illumina NovaSeq 6000.

### Lipid peroxidation assay

The levels of lipid ROS were detected using lipid peroxidation probe C11-BODIPY 581/591 (Thermo Fisher, USA) by following the manufacturer’s instructions. Briefly, cells were incubated with 2.5 mM probe in the culture medium for 30 min at 37˚C in the dark. Confocal images were acquired using a laser scanning microscope (Nikon). The fluorescence intensity was quantified using Image-Pro Plus 6.0 software.

### Measurement of intracellular Fe^2+^

Iron assay kit (Nanjing Jian Chen Bioengineering Institute) was used following the manufacturer’s instructions.

### Knockdown and overexpression experiments

Establishing stable knockdown (KD) or overexpression (OE) cell lines using lentivirus transduction: three PLKO.1-shYTHDF1.puro vectors were constructed to knock down mouse *Ythdf1* mRNA (*EcoR*I/*Age*I, Thermo, USA) and PCDH-YTHDF1 (E*coR*I) vector was constructed to overexpress YTHDF1 (Table S1). To produce lentivirus particles, 293T cells were transfected with 1.5 μg of PSPAX2, 0.5 μg of PMD2.g, and 2.5 μg of sh-YTHDF1/OE-YTHDF1 plasmids using calcium phosphate method. The transfection medium was replaced with regular culture medium after 14 h and after 48 h, culture medium containing the lentivirus particles was collected, filtered (0.22 μm filter, SLGP033RB, Millipore), and stored frozen.

To infect HT22 cells, 400 μl of the viral medium was added in the presence of 4 μg/ml polybrene. At 48 h post-infection, the cells underwent selection with 2 μg/ml puromycin for 8–10 passages. To verify YTHDF1 expression changes western blots and immunofluorescence analysis were conducted using anti-YTHDF1 antibody (Proteintech, No. 17479–1-AP).

### siRNA interference

Small interfering RNAs (siRNAs) targeting *Mettl3* or *p53* (Table S2) were purchased from JTS scientific (Wuhan, China). Briefly, siRNA (100 pM) and 4 µl Lipo8000™ transfection reagent (Beyotime, China) was mixed in 125 μl Opti-MEM medium and added to HT22 cells in six-well plates (Gibco, USA) plated at density of 300,000 cells per well 24 h before transfection.

### AAV vector packaging and delivery

For adeno-associated virus (AAV)-mediated KD experiments in MCAO/R models, a custom pAAV9 viral vector carrying shRNAs targeting mouse YTHDF1 (5'- GCTGAAGATTATCGCTTCCTA-3'was purchased from KEL Biotech (Shanghai). The sh*Ythdf1* vector contains a neuron-specific promoter (hSyn) and an enhanced-green fluorescence protein (EGFP) reporter gene. Three weeks before MCAO/R, 1 μL of AAV vector (1.00E + 13 vg) was stereotaxically injected into the right ventricle at bregma: −0.5 mm, interaural line: −1.1 mm, and cranium: −3.0 mm at the rate of 0.2 μL/min. Three weeks after the administration of pAAV9-hSyn-EGFP-shRNA, the EGFP expression from the AAV construct was confirmed using IVIS fluorescence imaging. 17 mice (AAV-NC n = 9; AAV-shYTHDF1 n = 8) were used for MCAO/R. Three mice died after the surgery and five mice developed subarachnoid hemorrhage (two injected AAV-NC and three AAV-shYTHDF1), therefore were excluded from further analysis.

### RNA and protein analysis

#### RNA immunoprecipitation (RIP)

HT22 cells were lysed in Lysis buffer (APExBIO USA) supplemented with PMSF (MeilunBio, Dalian, China) and RNase inhibitor (GLPBIO USA). Magnetic beads (Thermo, USA) were pre-incubated with anti-YTHDF1 (3.5 μg; Proteintech, cat#17479–1-AP) or anti-IgG (3.5 μg; CST, cat#2729) overnight at 4 °C. Cell lysates were incubated with magnetic bead-antibody complexes while rotating for 3 h at 4 °C. After washing, total RNA was isolated from the beads using Trizol (TaKaRa, Japan).

#### mRNA and protein stability assays

2.5 mg/ml transcriptional inhibitor actinomycin D (ActD, Sigma Aldrich) or 50 μg/ml translation inhibitor cycloheximide (CHX) (Sigma Aldrich) was added to PLKO.1-NC or PLKO.1-YTHDF1 stable HT22 lines. The collection of cells was conducted at designated time points, including 0, 3, and 6 h. Total RNA was extracted for qRT-PCR analysis and lysates were obtained for western-blots.

#### Puromycylation and proximity ligation detection (Puro-PLA)

Experiments were performed as previously described. Cells were fixed in PFA-sucrose (4% formaldehyde, 4% sucrose in PBS-MC) for 15 min at room temperature, and permeabilized in PBTR (0.1% Triton X-100 in PBS) for 15 min. Following a 1 h incubation at 37 °C with Duolink blocking solution (Sigma Aldrich, USA), cells were incubated with primary antibodies diluted in Duolink antibody diluent for 1.5 h at 37 °C: anti-p53 (1:500; Proteintech, cat# 10,442–1-AP) and anti-puromycin (1:500, DSHB, CAT # PMY-2A4). After washing, PLA probes from Duolink in Situ FITC kits (Sigma Aldrich, cat# DUO92004, DUO92002, and DUO92014) were used followed by subsequent ligation and amplification steps. After the last wash, the cells were stained with DAPI (Sigma Aldrich, cat# duo82040-5 ml) and mounted on glass slides. In control experiments, cells were pre-treated with 60 μM anisomycin for 30 min at 37 °C.

#### Immunofluorescent staining (IF)

Mice were perfused with normal saline and 4% PFA. 20 μm brain sections were obtained using a freezing microtome (Servicebio, Wuhan). Subsequently, the brain sections were washed with PBS and permeabilized using 0.1% Triton X-100 in PBS. The sections were blocked with 5% goat serum in PBS 2 h and then incubated in the primary antibody solution overnight at 4 °C. Then the sections were incubated with the second antibody for 3 h at room temperature. The antibodies used in this study are shown in Table S5. DAPI (No. MA0128; MeilunBio) staining was performed and the coverslips were mounted. Confocal images were acquired with a laser scanning microscope (Nikon, Japan).

### Statistical analysis

All data were analyzed using Prism software (Version 6.01, GraphPad, La Jolla, CA, USA). Student's t-test with Welch's correction or Mann–Whitney U test was used. *P* < 0.05 was considered statistically significant, and data are shown as means ± s.e.m or means ± s.d.

## Results

### YTHDF1 protein expression positively correlates with I/R-induced neuronal injury pathway expression

**Fig. 1:**
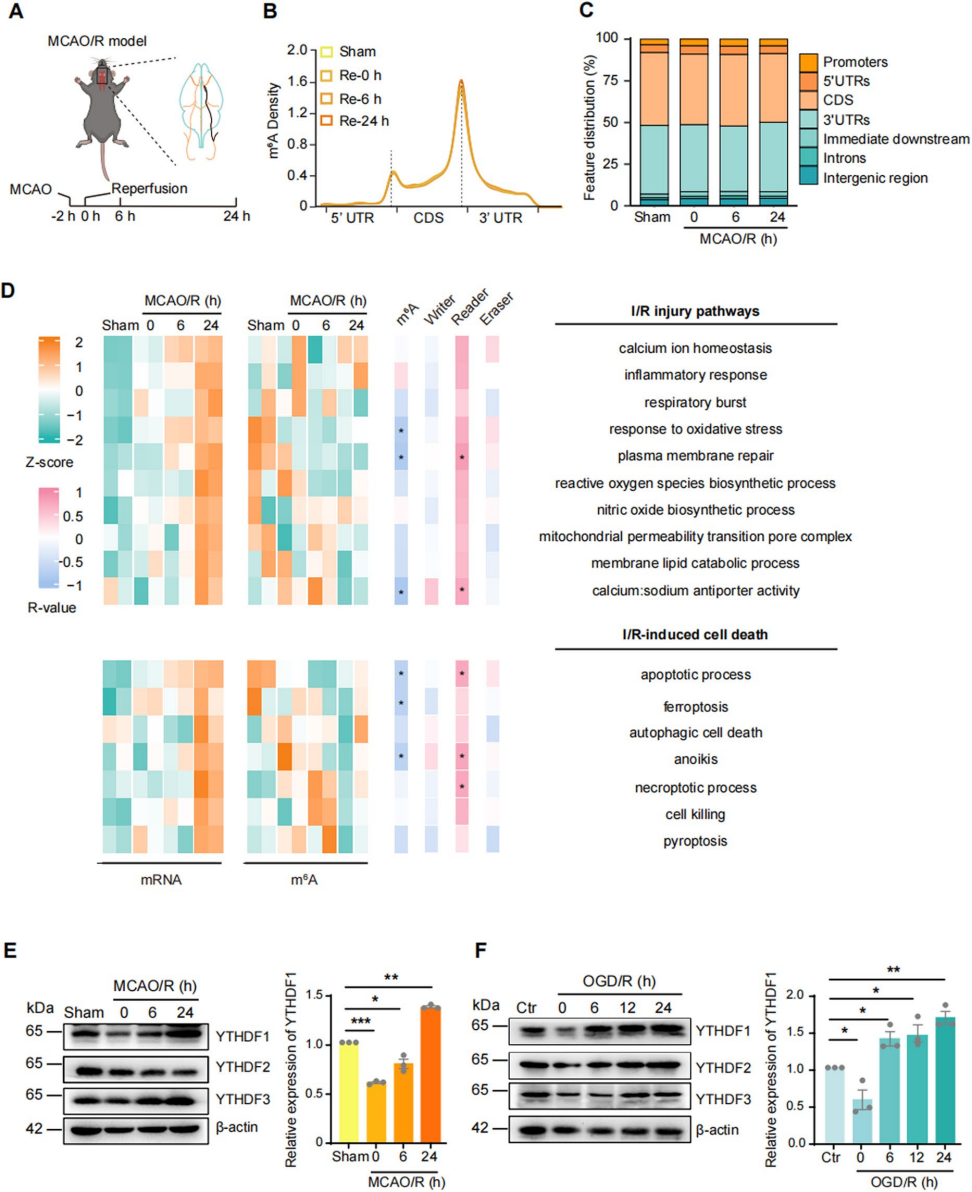
The expression of YTHDF1 was positively correlated with I/R injury-induced mRNA level changes of genes involved in I/R injury response pathways. (**A**) Schema of the MCAO/R surgical procedure. (**B**) The distribution of m^6^A sites along the length of mRNA transcripts in Sham and MCAO/R- 0/6/24 h groups at different time points. (**C**) Stacked bar plots showing the distribution of m^6^A peaks in different RNA regions in Sham and MCAO/R- 0/6/24 h groups at different time points. (**D**) Heat maps of changes in RNA abundance (left) and m^6^A modification levels (mid) in I/R injury pathways and I/R-induced cell death. Heatmap of correlation between RNA abundance and m^6^A modification levels, RNA abundance of m^6^A modification-related genes (right). (**E**) Western-blots of YTHDF1/2/3 proteins in peri-infarct tissue in Sham and MCAO/R-0/6/24 h groups. (**F**) Western-blots of YTHDF1/2/3 proteins in HT22 cells after OGD/R- 0/6/12/24 h groups

To explore the dynamic changes of m^6^A methylation, we mimicked cerebral I/R injury by inserting a nylon filament into the middle cerebral artery to induce occlusion for two hours followed by reperfusion (MCAO/R, Fig. 1A). Compared with Sham group, the neurological scores of MCAO/R-24 h mice were significantly higher, reflecting the severity of the injury-caused functional damage (*P* < 0.001; Sham: 0.167 ± 0.167, MCAO/R-24 h: 13.833 ± 0.601) (Supplementary Fig. 1A). The procedure caused cerebral infarct volume to increase in a time-dependent manner (*P* < 0.001; MCAO/R-0 h: 15.5 ± 1.50%; MCAO/R-6 h: 25.4 ± 1.28%; MCAO/R-24 h: 28.50 ± 0.93%) (Supplementary Fig. 1B). Total RNA was isolated from the peri-infarct tissue of MCAO/R-0/6/24 h mice and subjected to m^6^A Methylated RNA Immunoprecipitation Sequencing (MeRIP-seq). In each sample, we identified ~ 8 000 confident m^6^A peaks. Consistent with previous reports, the identified peaks were concentrated near the stop codon of the mRNAs (Fig. 1B). No significant changes of topological distribution were detected among different treatment groups (Fig. 1B, 1C). In the identified peak sequences, an enrichment of the consensus m^6^A motif RRACH (R = G or A; H = A, C, or U) was detected, supporting successful m^6^A peak identification (Supplementary Fig. 1 C).

I/R has been shown to induce an array of injury responses and cell death pathways, leading to neuronal cell death and neurological dysfunction (Zhang et al. 2022). We performed an ssGSEA (single-sample Gene Set Enrichment Analysis) analysis of the I/R-induced neuronal injury and cell death pathways (Fig. 1D, left orange-green gradient). The expression of the I/R injury pathway-related genes (e.g. calcium ion homeostasis, inflammatory response, respiratory burst, etc.) continuously increased during the 24 h acute response after MCAO/R, consistent with the existing literature (Zhang et al. 2022). Although the pathway-associated levels of m^6^A modifications changed dynamically following I/R (Fig. 1D, right; orange-green gradient), there was no corresponding increase in m^6^A levels. A correlation analysis between methylation changes and gene expression changes identified six significant negative correlations (*P* < 0.05, Student asymptotic *P*-value for correlation), indicating that hypermethylation of those pathway-associated mRNAs may promote their degradation and prevent excessive activities of these pathways (Fig. 1D, 1 st pink-blue gradient).

We further investigated the alterations in thirty-eight regulator mRNAs encoding m^6^A writers, readers, and erasers. Eleven regulator mRNAs were found to be differentially expressed (Supplementary Fig. 1D). The integrated writer expression exhibited negligible alterations during the I/R injury, whereas erasers surged at the MCAO/R-6 h but reverted to baseline level after 24 h. In contrast, the readers consistently increased after reperfusion (Supplementary Fig. 1E). Further analysis revealed that readers, but not writers or erasers, were positively correlated with the dynamic changes in five of the I/R-induced neuronal injury and cell death pathways (Fig. 1D, 2nd-4th pink-blue gradients). The results suggested that m^6^A readers are upregulated concurrently with the I/R injury pathway-associated genes.

**Fig. 2:**
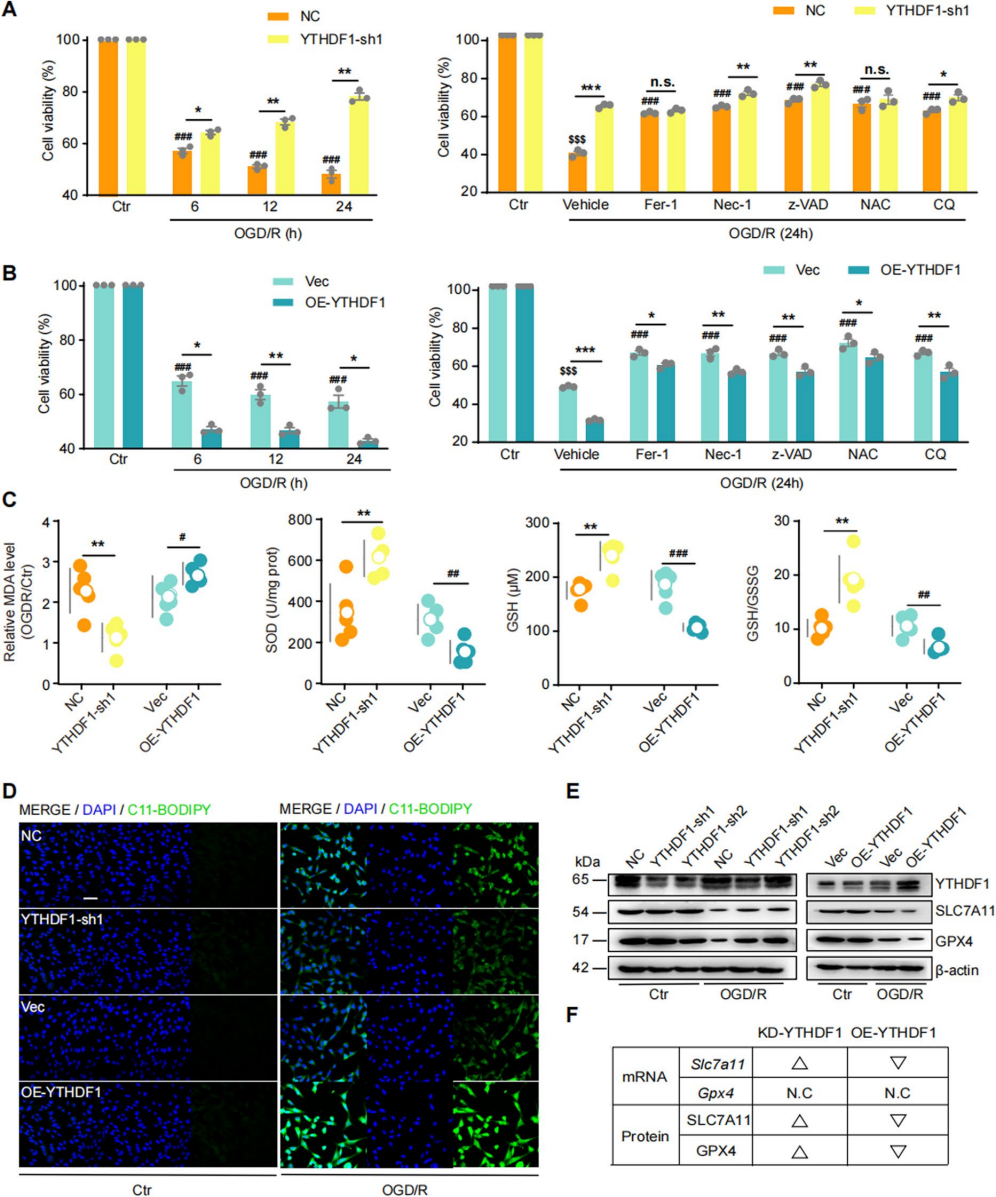
Inhibition or enhancement of YTHDF1 expression induced bidirectional changes in survival rate, oxidative stress level, and ferroptosis of HT22 cells after OGD/R. (**A**) Cell viability (normalized to Ctr groups) of HT22 cells transfected with NC or YTHDF1-sh1 at the control (Ctr) and OGD/R-6/12/24 h (left). Data were expressed as means ± s.e.m. *P < 0.05, **P < 0.01 versus NC group. ###*P* < 0.001 versus Ctr group. Cell viability in sh-YTHDF1 in control and OGD/R HT22 cells treated with, ferroptosis inhibitor/lipid peroxidation scavenger (Fer-1, 4 μM), necrosis inhibitor (Nec-1, 30 μM), apoptosis inhibitor (Z-VAD, 25 μM), ROS scavenger (NAC, 4 mM) and autophagy inhibitor (CQ, 10 μM) (right). Data were expressed as means ± s.e.m. *P < 0.05, **P < 0.01, ***P < 0.001 versus NC group. ###*P* < 0.001 versus Vehicle group. $$$*P* < 0.001 versus Ctr group. *n* = 3 experiments. (**B**) Cell viability of HT22 cells transfected with Vec or OE-YTHDF1 at the control and OGD/R-6/12/24 h (left). Data were expressed as means ± s.e.m. *P < 0.05, **P < 0.01 versus Vec group. ###*P* < 0.001 versus Ctr group. Cell viability in OE-YTHDF1 in control and OGD/R HT22 cells treated with Fer-1, Nec-1, Z-VAD, NAC and CQ (right). Data were expressed as means ± s.e.m. *P < 0.05, **P < 0.01, ***P < 0.001 versus Vec group. ###*P* < 0.001 versus Vehicle group. $$$*P* < 0.001 versus Ctr group. *n* = 3 experiments. (**C**) MDA level, SOD activity, GSH level and GSH/GSSG ratio in sh-YTHDF1 or OE-YTHDF1 in OGD/R HT22 cells. Data were expressed as means ± s.d (means: open circles; s.d: vertical bars to the leftside of the data points). *P < 0.05, **P < 0.01 versus NC group. #*P* < 0.05, ##*P* < 0.01, ###*P* < 0.001 versus Vec group. *n* = 5 experiments. (**D**) The C11-BODIPY 581/591 staining of lipid peroxidation in sh-YTHDF1 or OE-YTHDF1 in OGD/R HT22 cells. Scale bar = 50 μm. (**E**) Western-blots of YTHDF1, SLC7A11, and GPX4 proteins in sh-YTHDF1 or OE-YTHDF1 in OGD/R HT22 cells. (**F**) SLC7A11 and GPX4 expression in OGD/R treated KD or OE-YTHDF1 cells

The known cytoplasmic m^6^A reader YTHDF family includes YTHDF1, YTHDF2, and YTHDF3 (Xu et al. 2024). Hence, we quantified their protein expression dynamics *in vivo* using the MCAO/R model in mice and *in vitro* using the OGD/R model in HT22 cells. Neuron-enriched m^6^A reader YTHDF1 gradually increased in both MCAO/R and OGD/R models, while YTHDF2 protein had no significant changes. YTHDF3 expression showed similar changes to YTHDF1 in the MCAO/R model but not in the OGD/R model (Fig. 1E, 1 F; Supplementary Fig. 1 F, G). Together, these results demonstrate that I/R triggers increased neuronal expression of YTHDF1, which is positively correlated with the I/R-induced expression of genes associated with the neuronal injury pathway.

### Increased YTHDF1 promotes oxidative stress and ferroptosis in OGD/R

To explore the function of increased YTHDF1 expression during I/R injury, we generated HT22 cell lines stably expressing shYTHDF1 (KD-YTHDF1, KD: knockdown) or YTHDF1 cDNA (OE-YTHDF1, OE: overexpression) using lentiviral infection and puromycin selection. Three short hairpin RNAs targeting YTHDF1, YTHDF1-sh1/sh2/sh3, were tested and all attenuated YTHDF1 expression (Supplementary Fig. 2 A) while OE-YTHDF1 enhanced YTHDF1 expression (Supplementary Fig. 2B). Negative control (NC) cells were transduced with an shRNA targeting no known mammalian genes and puromycin selected. Compared to NC cells, KD-YTHDF1 cells were more viable after OGD/R treatment (Fig. 2A left). Conversely, OE-YTHDF1 cells were less viable after OGD/R treatment compared to Vec cells (HT22 cells stably expressing empty vector) (Fig. 2B left).

Next, we investigated cell death and injury pathways underlying the cell viability changes. We subjected KD-YTHDF1 and OE-YTHDF1 cells to three cell death pathway-specific inhibitors during OGD/R: Ferrostatin-1 (Fer-1, a ferroptosis inhibitor, 4 μM), Necrostatin-1 (Nec-1, a necrosis inhibitor, 30 μM), and z-VAD-FMK (z-VAD, a pan apoptosis inhibitor, 25 μM), and two injury pathway-specific inhibitors: N-Acetyl-L-cyteine (NAC, an ROS scavenger, 4 mM) and Chloroquine (CQ, an autophagy inhibitor, 10 μM) (Fig. 2A right). At the tested concentrations, the inhibitors did not cause any measurable effects on the cell viability of control, KD-YTHDF1, or OE-YTHDF1 cells under normal physiological conditions (Supplementary Fig. 2 C). All inhibitor treatments attenuated OGD/R-induced neuronal cell death albeit incompletely. Given that OGD/R-induced neuronal injury results from a composite effect of multiple cell death pathways, a single inhibition of any one of the pathways did not completely prevent cell death. The neuroprotective effect seen in the KD-YTHDF1 cells persisted in the presence of necrosis inhibitor Nec-1, apoptosis pan-inhibitor z-VAD, or autophagy inhibitor CQ, but disappeared when the ferroptosis inhibitor Fer-1 was applied (Fig. 2A right). In addition, increased cell viability in KD-YTHDF1 was completely masked by the ROS scavenger NAC, consistent with the role of ROS as a driver of ferroptosis (Fig. 2A right). These results suggest that the neuroprotective effect of reducing YTHDF1 during OGD/R is primarily manifested via oxidative stress and ferroptosis pathways. In contrast to KD-YTHDF1, the aggravated cell death by OE-YTHDF1 under OGD/R conditions persisted in all treatments, suggesting that the augmented YTHDF1 expression can promote multiple cell death pathways, which cannot be prevented by inhibitors targeting single pathways (Fig. 2B right). Next, we evaluated oxidative stress and cellular products associated with ferroptosis. OGD/R-induced lipid peroxidation product of malondialdehyde (MDA), superoxide dismutase activity (SOD), glutathione (GSH), glutathione disulfide (GSSG), and GSH/GSSG ratio were affected by KD- or OE-YTHDF1. KD-YTHDF1 reduced MDA level, increased SOD activity, increased GSH and GSH/GSSG ratio whereas OE-YTHDF1 had the opposite effects (Fig. 2C). These results further support a dose-dependent effect of YTHDF1 in regulating oxidative stress and ferroptosis during OGD/R.

We further confirmed the effect of YTHDF1 on lipid peroxidation using C11-BODIPY, a lipophilic fluorescent dye that exhibits enhanced red fluorescence when reacting with lipid oxidants. We found that OGD/R-induced lipid peroxidation was attenuated in KD-YTHDF1 cells and exacerbated in OE-YTHDF1 cells (Fig. 2D and Supplementary Fig. 2D). This cell phenotype indicates ferroptosis, and prompted us to test the expression of SLC7A11 and GPX4 in KD- and OE-YTHDF1 cells. The SLC7A11/GSH/GPX4 pathway constitutes the primary anti-ferroptosis defense mechanism, which has been shown to be compromised during I/R, leading to enhanced ferroptosis. We found that OGD/R-induced reduction of SLC7A11 at both mRNA and protein levels, and of GPX4 at the protein level, was normalized in KD-YTHDF1 cells. Conversely, in OE-YTHDF1 cells, the decrease of SLC7A11 and GPX4 upon OGD/R was further intensified (Fig. 2E, F and Supplementary Fig. 2E-G). To further confirm the effect of YTHDF1 expression level on ferroptosis, we measured intracellular Fe^2+^ concentrations in OGD/R-treated HT22 cells. KD-YTHDF1 effectively inhibited, while OE-YTHDF1 exacerbated Fe^2+^ accumulation in HT22 cells (Supplementary Fig. 2H). In the absence of OGD/R treatment, neither SLC7A11/GPX4 expression nor intracellular Fe^2+^ accumulation was affected by YTHDF1 (Fig. 2E and Supplementary Fig. 2E-H).

Thus, increasing YTHDF1 expression induced by OGD/R exacerbates oxidative stress and ferroptosis, and preventing its increase is neuroprotective.

### p53 is a key target molecule of YTHDF1 in OGD/R

**Fig. 3:**
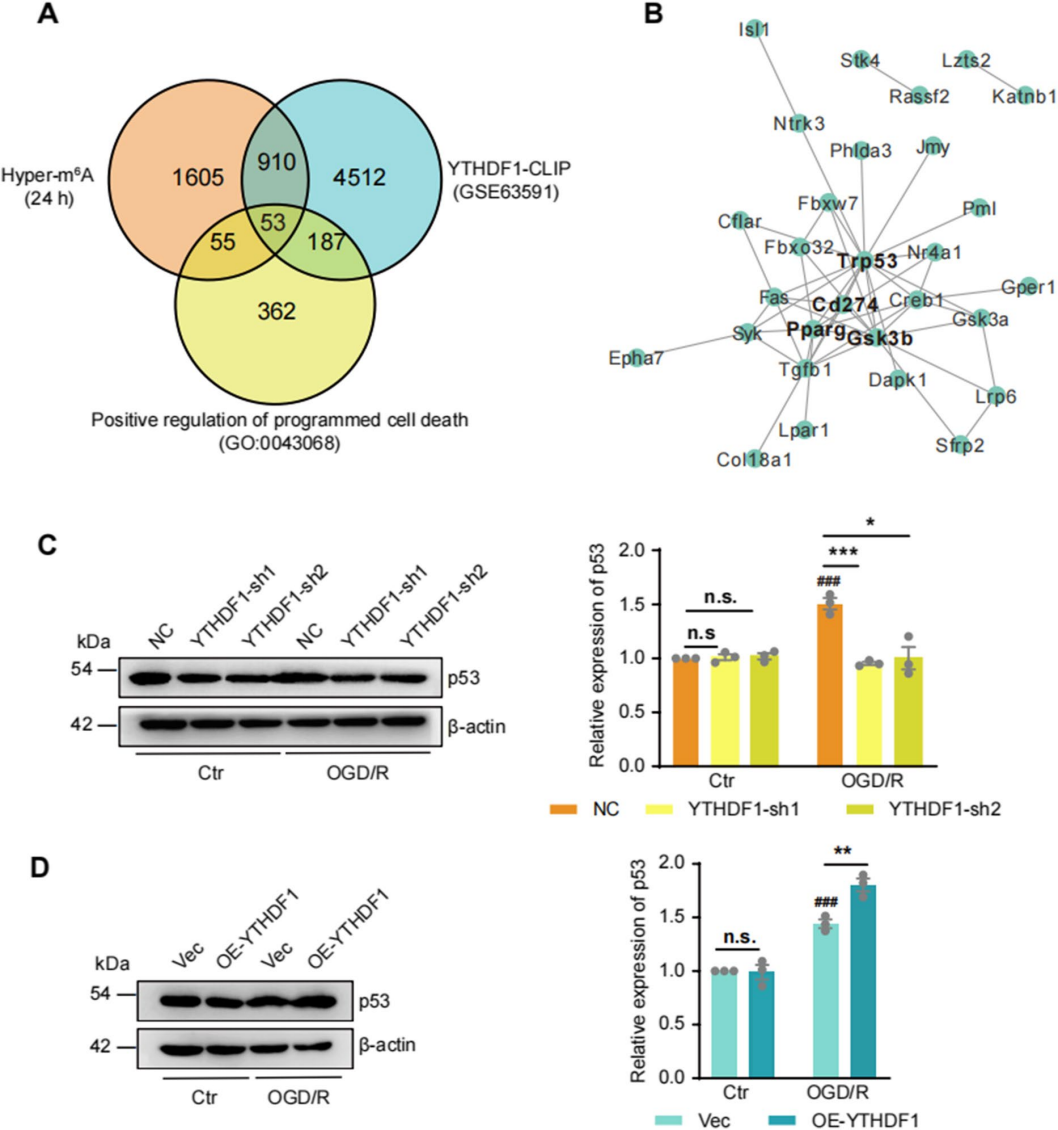
p53 is a key molecule in the regulation of I/R-induced neuronal damage by YTHDF1. (**A**) Venn diagram shows overlap of genes with YTHDF1 binding sites, increased m^6^A modification during the I/R phase, and involved in the nerve injury pathway caused by I/R. (**B**) PPI network of 29 genes with YTHDF1 binding sites, increased m.^6^A modification levels after I/R induction and involvement in I/R-induced injury pathway. (**C**) Western-blots and quantification of p53 protein in HT22 cells with KD-YTHDF1 and OGD/R. Data were expressed as means ± s.e.m. *P < 0.05, ***P < 0.001 versus NC group. ###*P* < 0.001 versus Ctr group. n = 3 experiments. (**D**) Western-blots and quantification of p53 protein in HT22 cells with OE-YTHDF1 and OGD/R. Data were expressed as means ± s.e.m. **P < 0.01 versus Vec group. ###*P* < 0.001 versus Ctr group. *n* = 3

How does the increased YTHDF1 in I/R promote oxidative stress and ferroptosis? Neither *Slc7a11* nor *Gpx4* mRNA was hypermethylated after MCAO/R (Supplementary Fig. 3 A), indicating no enhanced binding by the increased YTHDF1 protein. To identify potential YTHDF1 target mRNAs during I/R injury, we re-examined m^6^A expression dynamics in the peri-infarct tissue at three time points following MCAO/R (0, 6, 24 h, Supplementary Fig. 3B). Time series analysis grouped genes with similar m^6^A expression dynamics into a total of 14 clusters, out of which clusters 5, 7, and 14 showed significant m^6^A elevation following MCAO/R positively correlating with the changes in YTHDF1 expression and I/R injury-related gene pathway expression. 53 genes from these three clusters have been reported as YTHDF1 target mRNAs (Wang et al. 2015) (Fig. 3A), and belong to the “Positive regulation of programmed cell death” pathway (GO:0043068). A protein–protein interaction (PPI) network analysis revealed interactions among 29 genes, with p53 (also known as Trp53) as a central hub protein (Fig. 3B).

Consistent with the role of p53 in inducing cell death (Kuribayashi and El-Deiry 2008), p53 levels significantly increased over time following reperfusion in both MACO/R and OGD/R (Supplementary Fig. 3C-D). An inspection using Integrative Genomics Viewer (IGV) confirmed hypermethylation of *p53* mRNA in MCAO/R-24 h (Supplementary Fig. 4). Next, we tested p53 expression in KD-YTHDF1 and OE-YTHDF1 cells upon OGD/R, and observed decreased and increased p53 expression, respectively (Fig. 3C and D). Importantly, neither KD nor OE of YTHDF1 influenced p53 protein levels in the absence of OGD/R. These results suggest that the regulation of p53 expression by YTHDF1 depends on OGD/R.

### YTHDF1 promotes the translation of p53 in an m^6^A-dependent manner

**Fig. 4:**
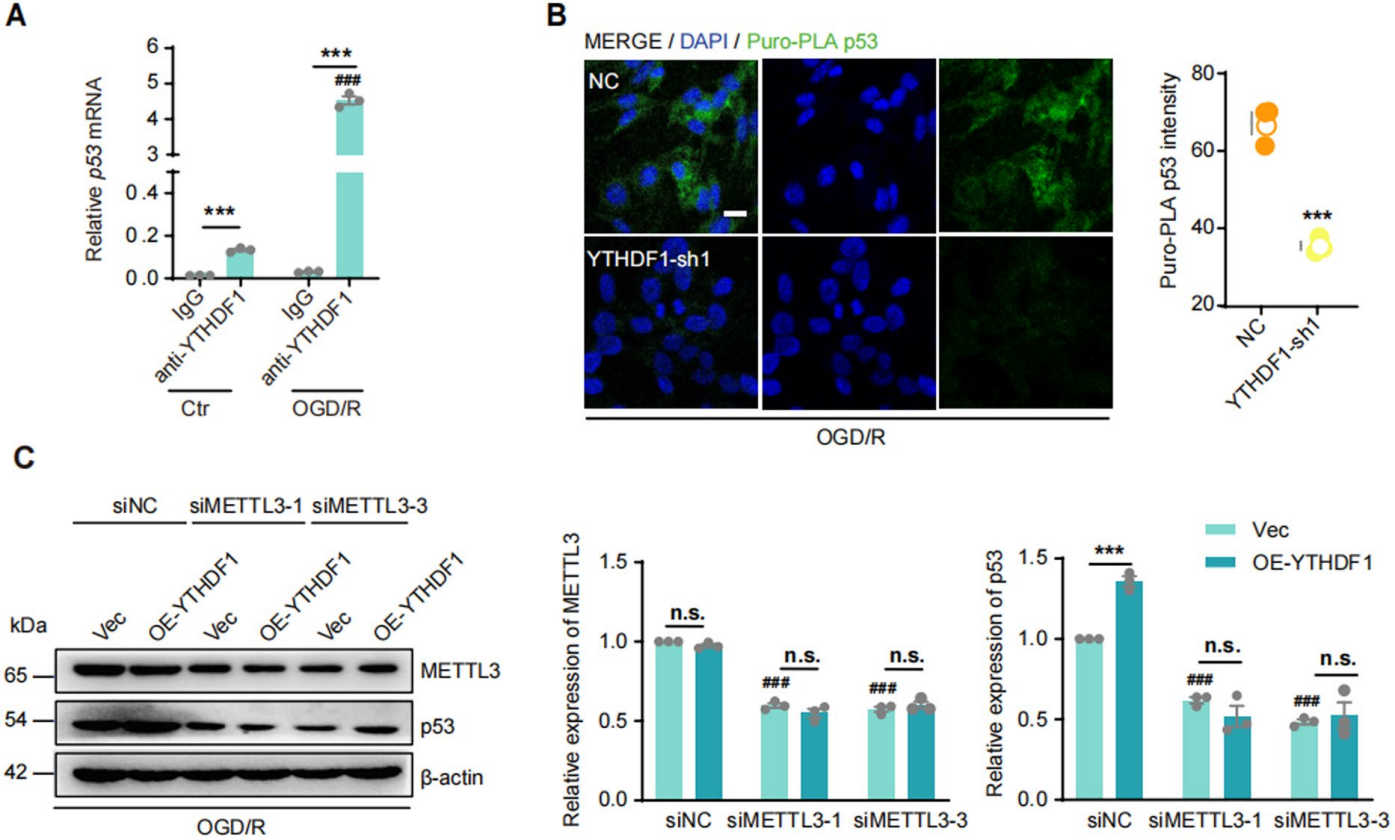
YTHDF1 promotes p53 translation after I/R in an m^6^A-dependent manner. (**A**) RIP detected the interaction between YTHDF1 and *p53* mRNA in control and OGD/R HT22 cells. Data were expressed as means ± s.e.m. ***P < 0.001 versus IgG group. ###*P* < 0.001 versus Ctr group. *n* = 3 experiments. (**B**) Puro-PLA images detected the p53 translation in HT22 cells with KD-YTHDF1 and OGD/R. Scale bar = 10 μm. Data were expressed as means ± s.e.m. ***P < 0.001 versus NC group. *n* = 3. (**C**) Western-blots and quantification of METTL3 and p53 proteins in HT22 cells with OE-YTHDF1 and OGD/R. Data were expressed as means ± s.e.m. ***P < 0.001 versus Vec group. ###*P* < 0.001 versus siNC group. *n* = 3 experiments

Next, we measured the binding between YTHDF1 and *p53* mRNA using RNA immunoprecipitation (RIP). Compared to physiological culture conditions, OGD/R increased the binding of YTHDF1 to *p53* mRNA by 34-fold (Fig. 4A). KD or OE of YTHDF1 had no effect on *p53* mRNA expression, *p53* mRNA stability, or the p53 protein degradation rate in either condition (Supplementary Fig. 5A-C), suggesting that the enhanced binding by YTHDF1 may specifically promote the translation of p53 protein.

To test this possibility, we applied p53 Puro-PLA to measure p53 translation upon OGD/R treatment (Fig. 4B, Supplementary Fig. 5D). After 10 min incubation of puromycin, anisomycin (a protein synthesis inhibitor) sensitive fluorescent p53 Puro-PLA puncta were detected in the cytoplasm of HT22 cells, representing newly synthesized p53 protein (Supplementary Fig. 5E). In KD-YTHDF1 cells following OGD/R treatment, p53 Puro-PLA signals were significantly reduced compared to NC cells (Fig. 4B).

To confirm that the YTHDF1-mediated p53 translation during OGD/R is m^6^A-dependent, siRNAs targeting the m^6^A methyltransferase METTL3 were transfected. Reduced METTL3 protein expression was verified by Western blotting (Supplementary Fig. 5 F). si-METTL3s blunted the p53 translational response in both Vec control and OE-YTHDF1 cells, indicating that YTHDF1 promotes p53 translation during I/R injury in an m^6^A-dependent manner (Fig. 4C; Supplementary Fig. 5G).

### p53 mediates m^6^A/YTHDF1 effect on oxidative stress and ferroptosis

**Fig. 5:**
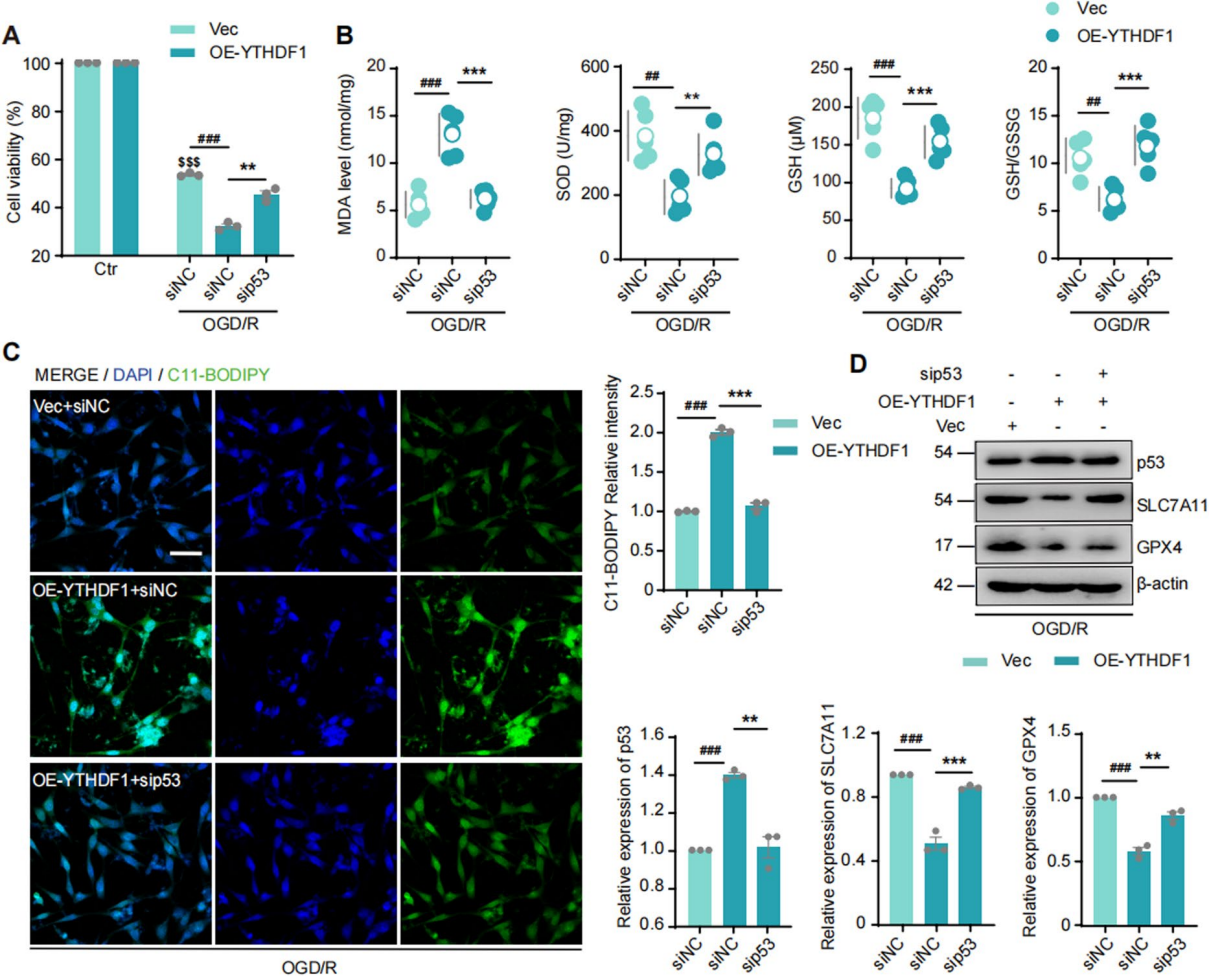
Inhibition of p53 reversed the regulation of YTHDF1 on the survival rate, oxidative stress level, and ferroptosis of HT22 cells after OGD/R. (**A**) Cell viability (normalized to Ctr groups) in OE-YTHDF1 HT22 cells with sip53 and OGD/R. Data were expressed as means ± s.e.m. **P < 0.01 versus siNC group. ###*P* < 0.001 versus Vec group. $$$*P* < 0.001 versus Ctr group. *n* = 3 experiments. (**B**) MDA level, SOD activity, GSH level and GSH/GSSG ratio in OE-YTHDF1 HT22 cells with sip53 and OGD/R. Data were expressed as means ± s.d. **P < 0.01, ***P < 0.001 versus siNC group. ##*P* < 0.01, ###*P* < 0.001 versus Vec group. n = 5 experiments. (**C**) The C11-BODIPY 581/591 staining of lipid peroxidation in OE-YTHDF1 HT22 cells with sip53 and OGD/R. Scale bar = 50 μm. Data were expressed as means ± s.e.m. ***P < 0.001 versus siNC group. ###*P* < 0.001 versus Vec group. *n* = 3 experiments. (**D**) Western-blots of YTHDF1, SLC7A11, and GPX4 proteins in OE-YTHDF1 HT22 cells with sip53 and OGD/R. Data were expressed as means ± s.e.m. **P < 0.01, ***P < 0.001versus siNC group. ###*P* < 0.01 versus Vec group. *n* = 3 experiments

To test whether p53 is necessary for the m^6^A/YTHDF1 effect on oxidative stress and ferroptosis, we applied Pifithrin-α (PFT-α), a p53 transcription inhibitor, to OE-YTHDF1 cells. PFT-α effectively rescued OGD/R-induced neuronal death (Supplementary Fig. 6 A). Similar to PFT-α, sip53 RNA transfection (Supplementary Fig. 6B) also improved the survival of OE-YTHDF1 cells (Fig. 5A). These results indicate that p53 is a crucial mediator of m^6^A/YTHDF1 function in I/R neuronal injury.

We then evaluated the impact of sip53 on MDA, SOD, GSH, GSH/GSSG ratio, and lipid peroxidation. sip53 significantly alleviated oxidative stress and lipid peroxidation in OGD/R-treated OE-YTHDF1 cells (Fig. 5B, C). Furthermore, the suppression of SLC7A11 and GPX4 in OE-YTHDF1 cells was alleviated by the p53- targeting siRNA (Fig. 5D, Supplementary Fig. 6 C). These results suggest that p53 plays an essential role in mediating YTHDF1 regulation of oxidative stress and ferroptosis during I/R injury.

### AAV-shYTHDF1 alleviates MCAO/R injury in mice

**Fig. 6:**
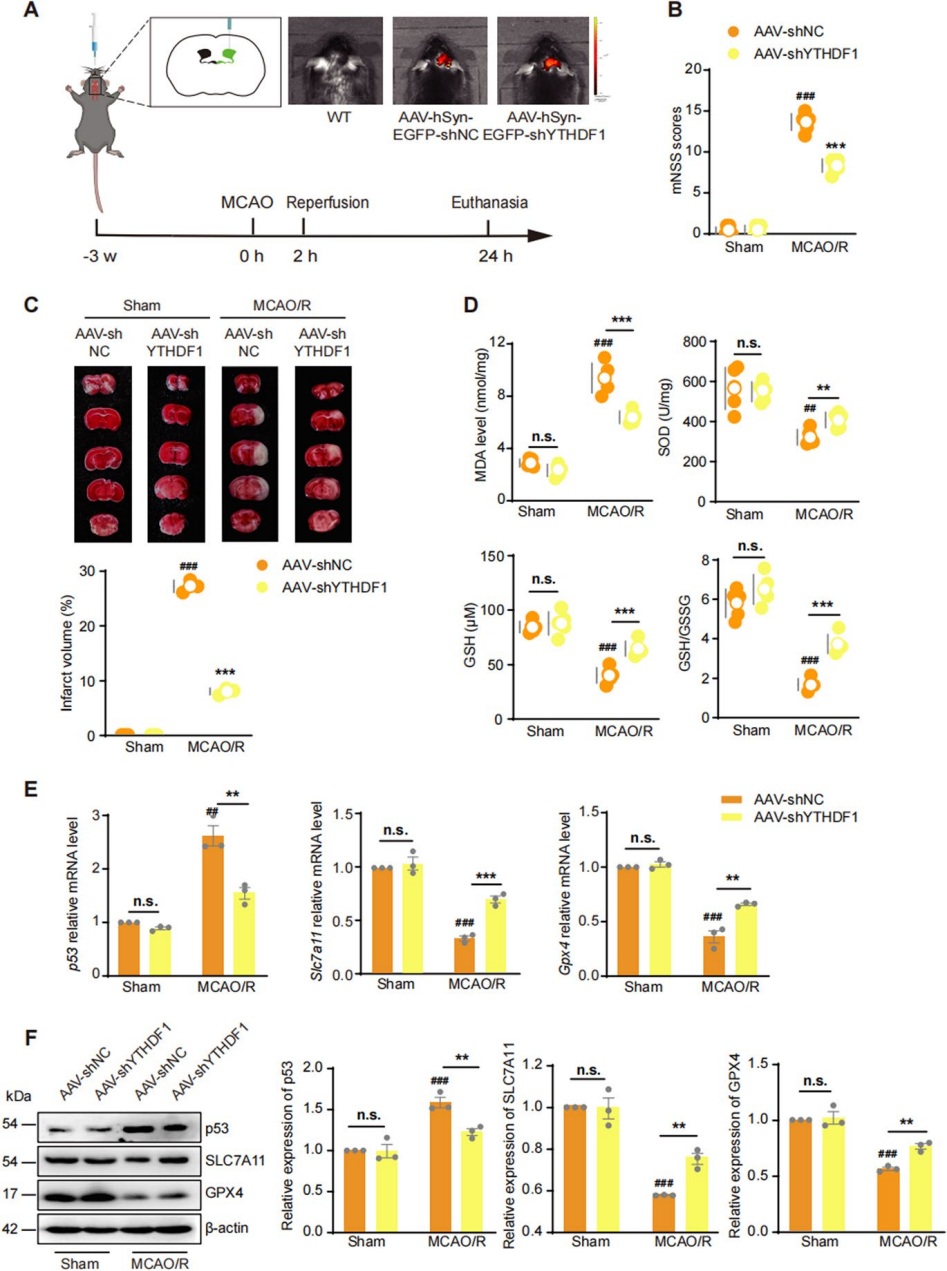
Specific knockdown of YTHDF1 expression in neurons alleviate MCAO/R-induced nerve injury in mice (**A**) Schematic of AAV-hSyn-EGFP-shYTHDF1 administration and analysis. (**B**) mNSS scores were used to evaluate neurological impairment in AAV-shNC and AAV-shYTHDF1 mice post-MCAO/R. Data were expressed as means ± s.e.m. ***P < 0.001 versus AAV-shNC group, ###*P* < 0.001 versus Sham group, *n* = 6 animals in each group. (**C**) TTC stained brain slices to evaluate cerebral infarct volume and infarct volume quantification in AAV-sh-YTHDF1 mice post-MCAO/R. Data were expressed as means ± s.e.m. ***P < 0.001 versus AAV-shNC group. ###*P* < 0.001 versus Sham group. n = 3 animals in each group. (**D**) MDA level, SOD activity, GSH level and GSH/GSSG ratio in AAV-sh-YTHDF1 mice post-MCAO/R. Data were expressed as means ± s.d. **P < 0.01, ***P < 0.001 versus AAV-shNC group. ##*P* < 0.01, ###*P* < 0.001 versus Sham group. n = 5 animals in each group. (**E**) Quantification of *p53*, *Slc7a11* and *Gpx4* mRNA in AAV-sh-YTHDF1 mice post-MCAO/R. Data were expressed as means ± s.e.m. **P < 0.01, ***P < 0.001 versus AAV-shNC group. ###*P* < 0.001 versus Sham group. *n* = 3 animals in each group. (**F**) Western-blots of YTHDF1, SLC7A11, and GPX4 proteins in AAV-sh-YTHDF1 mice post-MCAO/R. Data were expressed as means ± s.e.m. **P < 0.01 versus AAV-shNC group. ###*P* < 0.001 versus Sham group. *n* = 3 animals in each group

To test the potential neuroprotective effect of KD-YTHDF1 during I/R injury in vivo, we used an miR30-based shRNA expression system and constructed hSyn-EGFP-miR30 5’-shYTHDF1-miR30 3’ AAV virus to specifically knock down YTHDF1 in neurons. AAV virus was delivered into the right cerebral ventricle of 4 weeks-old male mice and MCAO/R was conducted at 7 weeks of age. Successful viral transduction was confirmed by detecting EGFP fluorescence using an in vivo spectrum imaging system (Fig. 6A). A non-targeting shRNA (shNC) was used as control. Co-immunostaining of cortical sections with YTHDF1 validated successful neuron-specific YTHDF1 reduction (Supplementary Fig. 7). Compared to the mice that received AAV-shNC, the mice that received AAV-shYTHDF1 injection had significantly reduced neurological impairment and infarct volume after MCAO/R (Fig. 6B and C). Consistent with the in vitro results, injection of AAV-shYTHDF1 mitigated MCAO/R-induced oxidative stress as measured by MDA, SOD, GSH, and the GSH/GSSG ratio in the peri-infarct tissue (Fig. 6D) and had normalized expressions of p53, SLC7A11, and GPX4 (Fig. 6E and F). Thus, the in vivo knockdown of YTHDF1 in neurons effectively prevented p53 upregulation, oxidative stress, and ferroptosis induction, thus significantly alleviating the MCAO/R-induced neuronal injury in mice.

## Discussion

3.3 million people die from ischemic stroke annually (by World stroke organization-Global Stroke Fact Sheet 2022). Alleviating neuronal cell death and extending the therapeutic window of revascularization are crucial for intervention development but clinically effective cerebroprotective agents that can be used prior to and during recanalization remain elusive. Stroke Preclinical Assessment Network (SPAN) has designed and implemented more rigorous, multi-laboratory preclinical trials to evaluate candidate interventions and identified uric acid, a potent antioxidant, to exceed the efficacy boundary and warrant future human studies (Lyden et al. 2023). Recently, LK-2, a compound targeting the glutamate-binding site on acid-sensing ion channels has been identified to effectively reduce the infarct volume and improve sensorimotor recovery in mice models of ischemic stroke (Lai et al. 2024).

The efficacy of targeting RNA modification pathways in treating ischemic stroke remains untested and the role of m^6^A in the expression and activation of cell survival/damaging mechanisms has been controversial (Han 2024; Xu et al. 2024). Our study reveals a robust m^6^A-dependent, YTHDF1-mediated mechanism to promote p53 expression and ferroptosis, leading to neuronal cell death during I/R injury. The effect is consistent with multiple publications in m^6^A preclinical animal models and in human patients wherein m^6^A increases after I/R and hypermethylation contributes to oxidative stress and cell death pathways (Shao et al. 2022; Xu et al. 2020a; Yi et al. 2021; Zhu et al. 2023). The mechanism further elucidates an oxidative stress-dependent ferroptosis pathway highly relevant to I/R (Bu et al. 2021; Guan et al. 2019; Guo et al. 2023; Xu et al. 2023b). Our results are supported by evidence from a completely independent study, which assessed the protective effect of salvianolic acid C (SAC), an active ingredient in *Salvia miltiorrhiza*, against cerebral ischemia reperfusion injury (Guo et al. 2024; Shen et al. 2022). Although the authors attributed the protective effect of SAC to suppressing microglial polarization and promoting angiogenesis, SAC was identified in a separate study by Zou et al. as a selective inhibitor of YTHDF1 (Zou et al. 2023). These results are in contradiction with a recent study showing that I/R injury was worsened in the germline YTHDF1 knockout male mice, but not in female mice (Chokkalla et al. 2024). What causes the phenotypic differences is unclear but we can speculate that dose differences, compensatory mechanisms, differences in temporal and spatial expression among other effects of the different approaches may have led to the different experimental outcomes. The relationship between the m^6^A reader and the cellular functions may be heterogeneous and context-dependent. Under physiological conditions, YTHDF1 plays an important role for learning and memory function, axon development, and synapse development (Broix et al. 2025; Merkurjev et al. 2018; Shi et al. 2018; Zhuang et al. 2019); under pathological conditions such as traumatic brain injury or I/R, multifaceted roles of YTHDF1 have been associated with cellular responses such as inflammation and programmed cell death (Chen et al. 2024; Li et al. 2022).

p53 is well-established as a pro-apoptotic factor in I/R injury, and as a trigger for neuroinflammation and ferroptosis (Gao et al. 2023; Xu et al. 2023a; Zhao et al. 2021). In p53 KO mice, I/R injury was mitigated by the p53/PRAS40/mTOR pathway (Zhao et al. 2021). p53 promotes neuronal apoptosis also by binding and activating *Bax*, a pro-apoptotic protein that inhibits its anti-apoptotic counterpart Bcl-2/Bcl-XL (Yan et al. 2021). Silencing METTL3 alters the expression of RNA splicing variants that affect p53 signaling pathway and induces apoptosis (Dominissini et al. 2012). Moreover, YTHDF1’s role in promoting I/R-triggered translation of p53 is consistent with its function in promoting both apoptosis and ferroptosis during ischemic stroke. Notably, activation of p53 does not induce ferroptosis; but increases the likelihood by downregulating the SLC7A11/GPX4 anti-ferroptosis pathway (Jiang et al. 2015). In addition to *p53*, we also identified a hypermethylated peak in the nuclear receptor coactivator 4 (*Ncoa4*) mRNA, whose protein product is involved in the degradation of ferritin, thus releasing free iron into the cytosol and inducing ferroptosis (Hou et al. 2016). Whether the translation of NCOA4 during I/R is dependent on m^6^A and YTHDF1 is currently unknown.

YTHDF2 and YTHDF3 may play additional roles in I/R injury. Hou et al. showed that YTHDF2 enhanced oxidative stress response during cerebral I/R injury by binding to and promoting the degradation of *Nrf2* mRNA (Hou et al. 2023). YTHDF2 upregulation in myocardial I/R injury also aggravated I/R-induced ferroptosis by degrading *Slc7a11* mRNA in an m^6^A-dependent manner (Pang et al. 2023). In our study, although *Ythdf2* mRNA levels increased, its protein expression remained unaltered. Our data do not exclude YTHDF2 from further repressing SLC7A11 protein expression through degrading the mRNA. The role of YTHDF3 in I/R injury models remains elusive. A recent study of blood samples of stroke patients predicted YTHDF3 as one of the seven key m^6^A regulators in the immune response (Shen and Yue 2024). In our study, YTHDF3 increased at 24 h post-MCAO/R in vivo. But unlike YTHDF1, its expression remained unaltered in the OGD/R-treated HT22 cells. The difference may reflect cell type-dependent YTHDF3 regulation. Evidence shows that the m^6^A pathway in astrocytes and microglia contributes to the pathophysiology of ischemic stroke (Xu et al. 2024). YTHDF1 in microglia has been previously reported to be involved in inflammatory response mechanisms during the subacute phase of I/R injury (Zheng et al. 2020). Enhancing YTHDF1 expression worsened cerebral injury by promoting PTEN stability and facilitating *p65* mRNA translation (Li et al. 2022; Zheng et al. 2020). In HT22 cells subjected to OGD/R, YTHDF1 enhanced the stability of STAT5 in an m^6^A-dependent manner, thereby increasing endoplasmic reticulum (ER) stress-mediated neuroinflammation and apoptosis (Liu et al. 2024). Recent studies have highlighted YTHDF1/3 complex and m^6^A in modulating the translation of tau and calmodulin during neurodevelopment (Huang et al. 2022). The potential molecular complex through which YTHDF1 exerts its effects remains elusive and warrants further investigation.

More m^6^A regulators have been shown to modulate I/R response. The forced expression of YTHDC1, a nuclear m^6^A reader protein, decreased brain infarct volume and promoted neuronal cell survival by facilitating Akt phosphorylation through degrading *Pten* mRNA (Zhang et al. 2020), demonstrating an opposite role to YTHDF1. The counteracting functions of the reader proteins may contribute to the fine-tuning of cell response pathways during stroke, although the precise mechanisms of the gene expression response refinement in the affected regions remain unclear.

Remarkably, the effect of YTHDF1 to exacerbate ferroptosis can be specific to cell types. YTHDF1 in nucleus pulposus cells was shown to suppress ferroptosis by promoting the expression of SLC7A11 and GPX4 (Lu et al. 2024). YTHDF1 in prostate cancer was also found to repress the T cell-mediated antitumor immunity and ferroptosis (Wang et al. 2024). However, in hepatic stellate cells, KD-YTHDF1 prevented BECN1 plasmid-induced ferroptosis, presenting evidence consistent with the role of YTHDF1 in this study (Shen et al. 2021). The pleiotropic effects of YTHDF1 on ferroptosis may be attributed to the specific RNA and m^6^A expression profiles in different cell types and contexts. Thus, the molecular role of m^6^A regulators may not be separable from the model systems and their associated target RNAs.

We do not yet fully understand the gene expression regulatory mechanisms by dynamic m^6^A signals during I/R injury. The interpretation of the current study is limited because of the lack of information regarding the stoichiometry of the modification sites, which obscure the accuracy of data interpretation and functional prediction. We also lack evidence on the long-term effects of m^6^A dynamics in patients’ recovery. The METTL3 inhibitor STM2457 has been developed to target myeloid leukemia (Yankova et al. 2021). Given that silencing METTL3 attenuates OGD/R-induced neuronal cytotoxicity (Xu et al. 2020b), STM2457 may also have clinical benefits in stroke treatment. Recently, METTL3 has been shown to directly interact with p53 and amplify p53 stress responses by both catalytic-dependent and independent mechanisms (Raj et al. 2022). Further studies are required to elucidate the potential challenges and therapeutic benefits of targeting m^6^A. In future studies, sex, age, and comorbidities need to be carefully considered.

Taken together, our results suggest that reprogramming gene expression through dynamic m^6^A signal and its reader protein occurs in ischemic stroke. We reveal a key pathway mediated by *p53* mRNA hypermethylation and increased YTHDF1 expression to induce ferroptosis and exacerbate neuronal injury. However, demonstrating potentials in the future development of safer and efficacious clinical interventions for ischemic stroke requires a deeper understanding of the cellular and functional contexts of the underlying regulatory mechanisms.

## Supplementary Information

Below is the link to the electronic supplementary material.

Supplementary file1 (DOCX 33.1 MB)

## Data Availability

The RIP- sequencing and MeRIP-sequencing data in this study have been deposited in the Gene Expression Omnibus database under accession code GSE247068. Raw data from Fig. 1–6 and Supplement Figures S2, S3, S5 and S6 were deposited on Mendeley at 10.17632/6t2w5y36hs.1.
