# Supplementary material for: YTHDF1 promotes p53 translation and induces ferroptosis during acute cerebral ischemia/reperfusion through m6A-dependent binding
Source: Cell Biol Toxicol. 2025 Jul 1;41(1):112. doi: 10.1007/s10565-025-10061-3 (PMC12213927; doi:10.1007/s10565-025-10061-3)
Supplement: Supplementary file 1 — Supplementary file1 (DOCX 33.1 MB) [file 10565_2025_10061_MOESM1_ESM.docx]

**Supplementary Information**

**YTHDF1 Promotes p53 Translation and Induces Ferroptosis during Acute Cerebral Ischemia/Reperfusion through m^6^A-dependent Binding**

Xinyu Chang^1^, Bingwu Li^1, 3^, Wanxu Huang^1^, Aixia Chen^1^, Shengmin Zhu^1^, Yueyang Liu^1^, Xiaoling Liu^1,^ ^*^; Jingyu Yang^2, *^; Dan Ohtan Wang^1,3,^ ^*^

^1^ Wuya College of Innovation, Shenyang Pharmaceutical University, Shenyang, PR China

^2^ Department of Pharmacology, Shenyang Pharmaceutical University, Shenyang, PR China

^3^ Biology Program, Science Division, New York University Abu Dhabi, Abu Dhabi, United Arab Emirates

^*^Corresponding authors.

ohtan.wang@nyu.edu (Dan Ohtan Wang);

yangjingyu@syphu.edu.cn (Jingyu Yang);

lxl91321@126.com (Xiaoling Liu)

**Supplemental Materials and Methods**

Animals and MCAO/R

Adult male C57BL/6J mice aged 4-8 weeks (18-25 g) were purchased from Changsheng Biotechnology (Liaoning, China) and housed in the mice facility with the temperature at 23±0.5℃, humidity at 55-60%, and a 12hr-light/12-hr dark cycle, with ad libitium access to food and water. Animals were acclimatized to the new environment for one week before any experiment.

Before surgery, 6-8 weeks old mice were randomly divided into 4 groups (n = 6/group): the sham operation group (sham group); Re-0 h group (MCAO 2 h, Reperfusion 0 h); Re-6 h group (MCAO 2 h, Reperfusion 6 h); Re-24 h group (MCAO 2 h, Reperfusion 24 h). Before surgery, the mice were anaesthetized using intraperitoneal injection of 1.25% Avertin (20 µL/g). After confirming anesthetization, both sides of the animal neck were shaved with an electric razor to expose the skin. The operation site was disinfected with 75% ethanol. Under sterile conditions, a median neck incision was made to expose the surgical field and the right common carotid artery (CCA), external carotid artery (ECA) and internal carotid artery (ICA). The CCA was temporarily occluded with an arterial clip, the ECA was permanently occluded with sutures, and the ICA was sutured distally at the bifurcation point. A small incision was then made between the permanent and temporary sutures of the CCA. A 0.23 mm diameter surgical nylon thread (143003, Huayang Biotech, Shenzhen, China) was inserted into the ICA to block blood flow at the origin of the middle cerebral artery. The nylon thread was removed after 2 h, and the cervical incision was sutured. The mice were then placed in a 37°C nursing box for recovery. The sham surgery group underwent the same procedures except for the insertion of the nylon thread. We initiated the experiments with fifty C57BL6/J mice, of which 8 mice died after the surgery and 7 mice developed subarachnoid hemorrhage. These mice were excluded from any further analysis.

**Modified neurological severity scores (mNSS)**

mNSS was used to evaluate the neurobehavioral performance of mice in MCAO/R-24 h. The measurement scores included motor tests (tail lift test and Neurobehavioral Longa Score), sensory function (placement test and proprioceptive test), balance function (balance beam test), reflex loss and abnormal movement evaluation, with a total score of 18. Scores of 13-18 were classified as severe, 7-12 as moderate, and 1-6 as mild.

Infarct size evaluation

After cervical dislocation, brains were dissected into five coronal slices (2 mm thickness) and incubated in 2% 2,3,5-triphenyltetrazolium chloride solution (TTC, Solarbio, Beijing, China) for 20 min at 37°C in the dark, followed by 24 hours fixation in 4% paraformaldehyde (PFA). The infarct area of each slice was analyzed using Image-Pro Plus 6.0 software and calculated as the cerebral infarct volume (%) using the following formula: (VC-VL) / (2 × VC) × 100% (VC: volume of the contralateral hemisphere; VL: volume of stained ipsilateral hemisphere).

Oxidative stress measurement

Lysates from peri-infarct cerebral cortex of mice (n=5/group) were prepared by RIPA buffer homogenization. The levels of superoxide dismutase (SOD), malondialdehyde (MDA), glutathione (GSH) and glutathione disulfide (GSSG) were measured using commercial kits (Jiancheng, Nanjing, China) according to the manufacturer's protocols.

**MeRIP-seq data processing and analysis**

The initial stage of the analysis entailed the trimming of raw reads by utilizing Trim Galore (version 0.6.7, https://www.bioinformatics.babraham.ac.uk/projects/trim_galore/) with the parameter ‘--paired’. This was followed by the alignment of the trimmed reads to the mouse genome GRCm39/mm39 using HISAT2 (version 2.2.1) with default parameters. Unmapped and multi-mapped reads were excluded using samtools. Counts for annotated genes were generated using feature Counts (version 2.0.1) with the GENCODE VM30 annotation file. The raw counts were normalized using the estimate Size Factors function of the DESeq2 (version 1.32.0) to identify differentially expressed genes in RNA-seq. R package clusterProfiler was used to perform functional enrichment analysis.

Exomepeak2 was used for m^6^A peak calling with p-value = 0.05 as cutoff, and the log_2_FC= 0.5 as cutoff. When identifying the differentially methylated regions, a fold change greater than 1.5 is considered. BEDTools was used to merge all m^6^A regions, and the bamCompare function of deepTools was used to perform RPKM normalization and calculate m^6^A abundance. cliProfiler (https://github.com/Codezy99/cliProfiler) was used to visualize the distribution of m^6^A sites along the mRNA.

Time series clustering was performed using the R package Mfuzz.

For single-sample Gene Set Enrichment Analysis (ssGSEA), enrichment scores were calculated using the GSVA package based on the TPM (Transcripts Per Million) measurements. Methylation status was calculated as TPM (IP) over TPM (INPUT). Heatmaps of RNA expression levels and ssGSEA scores were plotted using ComplexHeatmap.

Protein-protein interaction networks were mapped using the STRING database and displayed using Cytoscape. Proteins exhibiting no interactions with others were manually removed, leaving 29 proteins in the displayed network.

Cells and OGD/R Model

Mouse hippocampal neuron HT22 cells (iCell Bioscience, Shanghai, China) were cultured in Dulbecco's Modified Eagle's medium (DMEM, Gibco, USA) supplemented with 10% fetal bovine serum (FBS, Gibco, USA) and 1% penicillin-streptomycin (Solarbio, Beijing, China), maintained in a 37°C humidified atmosphere of 5% CO_2_ incubator.

To conduct OGD/R, cells were washed with PBS and incubated in D-glucose (-), sodium pyruvate (-) DMEM for 6 h at 37°C in an ischemic incubator containing 1% O_2_, 5% CO_2_ and 94% N_2_. After 6 hours, deprived medium was replaced with regular medium and cells were returned to the incubator with 95% air and 5% CO_2_ for 0, 6 and 24 h, respectively.

Cell viability assay

HT22 cells (5 × 10^3^/well) were plated in 96-well plates 24 h before OGD/R. According to the manufacturer's instructions of the CCK-8 kit (Meilune, Dalian, China), 10 μl CCK-8 reaction solution was added to each well and incubated at 37°C for 1 h. The optical density (OD) at 450 nm was measured using an absorbance meter (Molecular Devices).

Cell death pathway inhibitor treatments: N-Acetyl-1-Cyteine (NAC, an ROS scavenger, Sigma, Shanghai, China), Ferrostatin-1 (Fer-1, a ferroptosis inhibitor, Aladdin, Shanghai, China), chloroquine (CQ, an autophagy inhibitor, AbMole BioScience), necrostatin-1 (Nec-1, a necrosis inhibitor, AbMole BioScience) and Z-VAD-FMK (Z-VAD, an apoptosis inhibitor, AbMole BioScience) were added to the culture medium 1 h before OGD treatment respectively.

Western blots

Peri-infarct tissue or HT22 cells were lysed using RIPA buffer containing protease inhibitors (Beyotime, China). Protein concentrations were determined using the BCA protein assay kit (Beyotime, China). After SDS-PAGE electrophoresis, proteins were transferred to polyvinylidene difluoride (PVDF) membranes (Millipore, USA) and blocked in 5% bovine serum albumin solution for 2 h at room temperature. Proteins were incubated with specific primary antibodies overnight at 4°C (Antibodies are listed in Table S3). After washed three times in PBST (PBS containing Tween 20) and incubated with secondary antibodies conjugated to horseradish peroxidase for 3 h at room temperature, the membranes were developed using ECL (Meilune, Dalian, China) and imaged using Invitrogen I Bright FL1500 imaging system (Thermo, USA). Band intensities were analyzed using Image J software.

Quantitative real-time PCR (RT-qPCR)

Total RNA was extracted using TRIZOL reagent according to the manufacturer's protocol (TaKaRa, Japan) and used for cDNA synthesis using PrimeScriptTM RT Kit (TaKaRa, Japan). Quantitative PCR was performed using SYBR Green PCR Master Mix (TaKaRa, Japan) according to the manufacturer's instructions. *β-Actin* was used as an internal control, and the relative expression was calculated using the 2^-ΔΔCT^. The primers used in this study are listed in Table S4.

**Supplemental Tables**

**Table S1. List of primer sequences for constructing adenovirus YTHDF1 knockdown or overexpression vectors**

| Names | Sequences (5' to 3') | |
| --- | --- | --- |
| NC (negative control) | forward | CCGGTCAACAAGATGAAGAGCACCAACTCGAGTTGGTGCTCTTCATCTTGTTGTTTTTG |
|  | reverse | AATTCAAAAACAACAAGATGAAGAGCACCAACTCGAGTTGGTGCTCTTCATCTTGTTGA |
| YTHDF1-sh1 | forward | CCGGTGCTGAAGATTATCGCTTCCTACTCGAGTAGGAAGCGATAATCTTCAGCTTTTTG |
|  | reverse | AATTCAAAAAGCTGAAGATTATCGCTTCCTACTCGAGTAGGAAGCGATAATCTTCAGCA |
| YTHDF1-sh2 | forward | CCGGTGGGTTGATTGTTGCATCTTTACTCGAGTAAAGATGCAACAATCAACCCTTTTTG |
|  | reverse | AATTCAAAAAGGGTTGATTGTTGCATCTTTACTCGAGTAAAGATGCAACAATCAACCCA |
| YTHDF1-sh3 | forward | CCGGTGCCCACAGCTATAACCCTAAACTCGAGTTTAGGGTTATAGCTGTGGGCTTTTTG |
|  | reverse | AATTCAAAAAGCCCACAGCTATAACCCTAAACTCGAGTTTAGGGTTATAGCTGTGGGCA |
| OE-YTHDF1 | forward | GATTCTAGAGCTAGCGATGTCGGCCACCAGCGTG |
|  | reverse | ATCCGATTTAAATTCGAATTTTATTGTTTGTTTCGATTCTGTCTTTC |

**Table S2. List of siRNA target sequences**

| Names | Sequences (5' to 3') |
| --- | --- |
| si-NC | UAAUGUAUUGGAACGCAUA |
| si*p53*-1 | CCACUUGAUGGAGAGUAUU |
| si*p53*-2 | GAAUGAGGCCUUAGAGUUA |
| si*Mettl3*-1 | CCUCAGUGGAUCUGUUGUG |
| si*Mettl3*-2 | GCACAUCCUACUCUUGUAA |
| si*Mettl3*-3 | CCGCAAGAUUGAGUUAUUU |

**Table S3. List of antibodes used in Western-blots.**

| Antibodies | Identifier | Dilution rate | Source |
| --- | --- | --- | --- |
| YTHDF1 | No. 17479-1-AP | 1:2000 | Proteintech |
| YTHDF2 | No. 24744-1- AP | 1:2000 | Proteintech |
| YTHDF3 | No. sc-377119 | 1:500 | Proteintech |
| p53 | No. 60283-2-Ig | 1:10000 | Proteintech |
| SLC7A11 | No. T57046 | 1:1000 | Abmart |
| GPX4 | No. T56959 | 1:500 | Abmart |
| β-actin | AF7018 | 1:4000 | Affinity Biosciences |

**Table S4. List of primer sequences in RT-qPCR**

| Name | Sequence(5'to3') | |
| --- | --- | --- |
| *Ythdf1* (Mus) | forward | CACAGTGACTCCCTCAACAAG |
|  | reverse | AGGTGGTAACATCCCCAATCTT |
| *p53* (Mus) | forward | TGGAAGGAAATTTGTATCCCGA |
|  | reverse | GTGGATGGTGGTATACTCAGAG |
| *Slc7a11* (Mus) | forward | TGGCGGTGACCTTCTCTGA |
|  | reverse | ACAAAGATCGGGACTGCTAATGA |
| *Gpx4* (Mus) | forward | GATGGAGCCCATTCCTGAACC |
|  | reverse | CCCTGTACTTATCCAGGCAGA |
| *β-actin* (Mus) | forward | GCTATGTTGCTCTAGACTTCG |
|  | reverse | GGATTCCATACCCAAGAAGG |

**Table S5. List of antibodies used in immunofluorescent staining**

| Antibodies | Identifier | Dilution rate | Source |
| --- | --- | --- | --- |
| YTHDF1 | No. 17479-1-AP | 1:500 | Proteintech |
| Goat Anti-Rabbit IgG (H+L) TRITC-conjugated | #S0015 | 1:200 | Affinity Biosciences |

**Supplemental Figures and Figure Legends.**

**Supplemental Figure S1:**

**
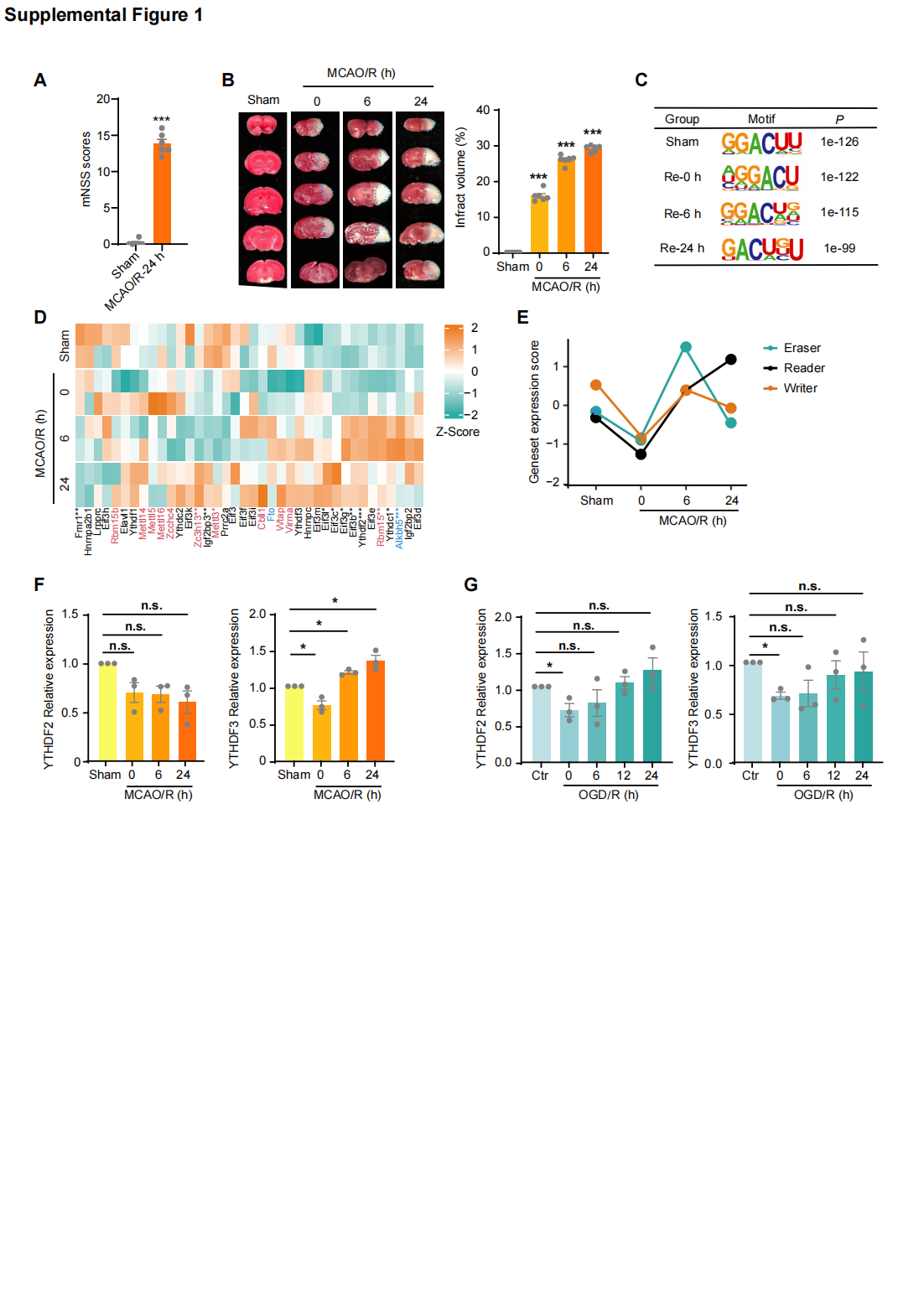
**

**Figure S1. Supplemental figure for Figure 1. Dynamic expression of m^6^A regulator proteins in the peri-infarct tissue of MCAO/R.** (A) mNSS behavioral scores were used to evaluate the neurological impairment in MCAO/R-24 h mice. Data were presented as means ± s.e.m. (****P*<0.001 versus Sham group, n=6 animals). (B) Representative images of 2, 3, 5-triphenyltetrazolium chloride (TTC) stained brain slices to evaluate cerebral infarct volume (enclosed areas represent infarction) and infarct volume quantification in MCAO/R-0/6/24 h mice. Data were expressed as means ± s.e.m. ****P*<0.001 versus Sham group, n=6 animals. (C) Top enriched 6nt-motifs within the m^6^A peaks in Sham and MCAO/R-0/6/24 h groups. (D) RNA expression Z-score of m^6^A reader (25 proteins), writer (11 proteins), and eraser (2 proteins) in Sham and MCAO/R-0/6/24 h groups. Asterisk (**P*<0.05, ***P*<0.01, or ****P*<0.001) next to the protein names indicate significant changes. (E) Gene Set Enrichment Analysis (GSEA) scores of m^6^A regulator expression in Sham and MCAO/R-0/6/24 h groups. (F) Western-blot quantification of YTHDF2/3 in Fig 1E. (G) Western-blot quantification of YTHDF2/3 in Fig 1F. Data were expressed as means ± s.e.m. * *P*<0.05, ** *P*<0.01, or ****P*<0.001 versus Sham group. n=3 animals in each group.

**Supplemental Figure S2:**

**
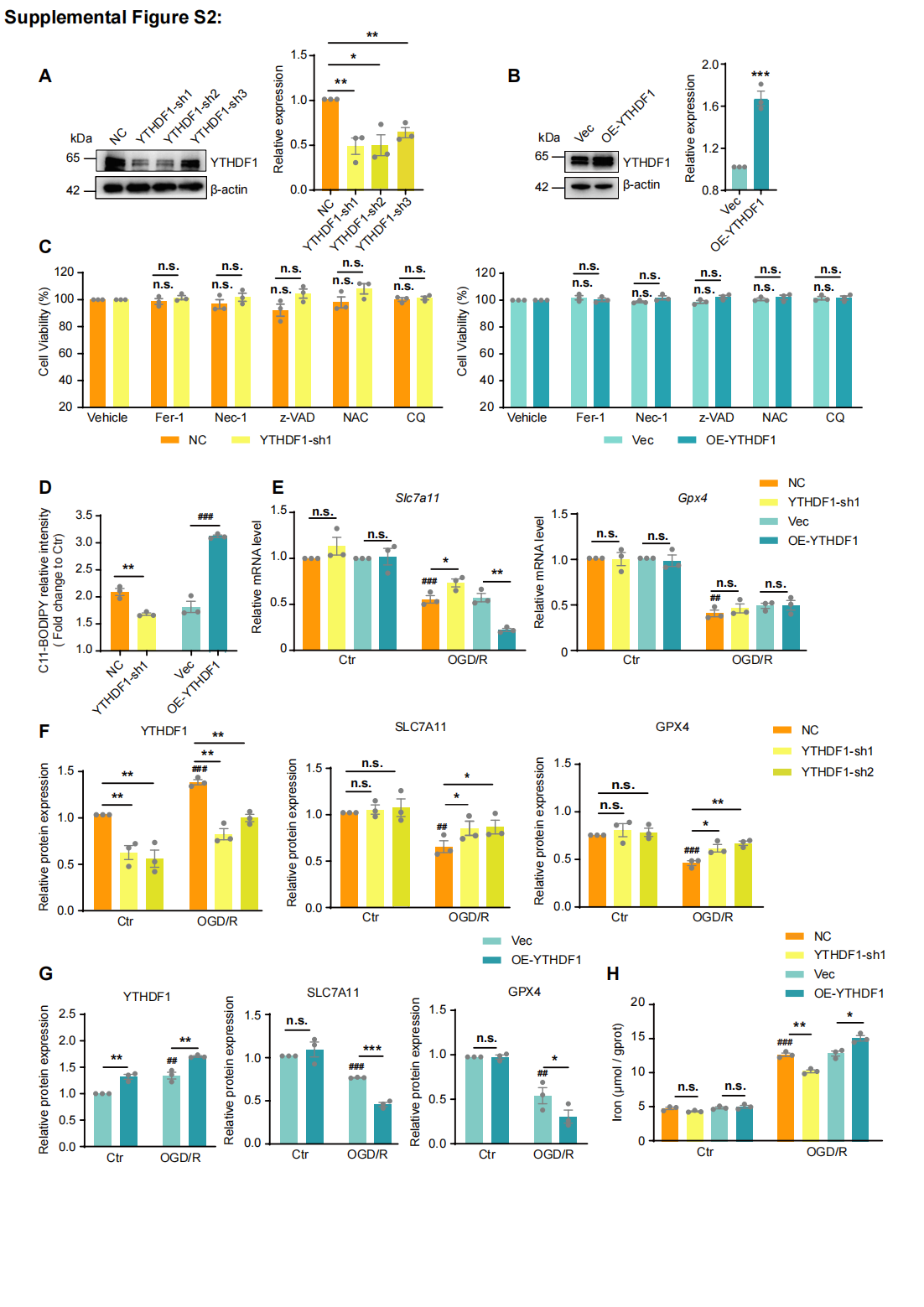
**

**Figure S2. Supplemental figure for Figure 2. Inhibiting or enhancing YTHDF1 expression induces bidirectional changes in SLC7A11 and GPX4 expression and ferroptosis pathway.** (A) Western-blots and quantification of YTHDF1 in HT22 transfected with NC or YTHDF1-sh1/sh2/sh3. Data were presented as means ± s.e.m. * *P*<0.05, ** *P*<0.01 versus NC group. n=3 experiments. All three shRNAs effectively reduced YTHDF1 expression. (B) Western-blots and quantification of YTHDF1 in HT22 transfected with Vec (empty vector) or OE-YTHDF1. Data were presented as means ± s.e.m. ****P*<0.001 versus Vec group. n=3 experiments. (C) Cell viability of HT22 cells treated with Fer-1, Nec-1, Z-VAD, NAC, and CQ upon KD-YTHDF1 or OE-YTHDF1. Data were expressed as means ± s.e.m. (n=3 experiments). (D) Quantification of lipid peroxidation detection experiments in Fig 2D. Data were expressed as means ± s.e.m. ***P*<0.05 versus NC group. ###*P*<0.001 versus Vec group. n=3 experiments. (E) Quantification of *Slc7a11* and *Gpx4* mRNA in KD-YTHDF1 or OE-YTHDF1 in control and OGD/R HT22 cells. Data were expressed as means ± s.e.m. * *P*<0.05, ** *P*<0.01 versus NC group or Vec group. ##*P*<0.01, ###*P*<0.001 versus NC + Ctr group. n=3 experiments. (F, G) Quantification of normalized YTHDF1, SLC7A11, and GPX4 expression in Fig 2E. Data are presented as means ± s.e.m. * *P*<0.05, ** *P*<0.01, *** *P*<0.001 versus NC group or Vec group. ##*P*<0.01, ###*P*<0.001 versus NC + Ctr group. n=3 experiments. (H) Quantification of Fe^2+^ in KD-YTHDF1 or OE-YTHDF1 HT22 cells subjected to OGD/R. Data were expressed as means ± s.e.m. * *P*<0.05, ** *P*<0.01 versus NC group or Vec group. ###*P*<0.001 versus NC + Ctr group. n=3 experiments.

**Supplemental Figure S3:**


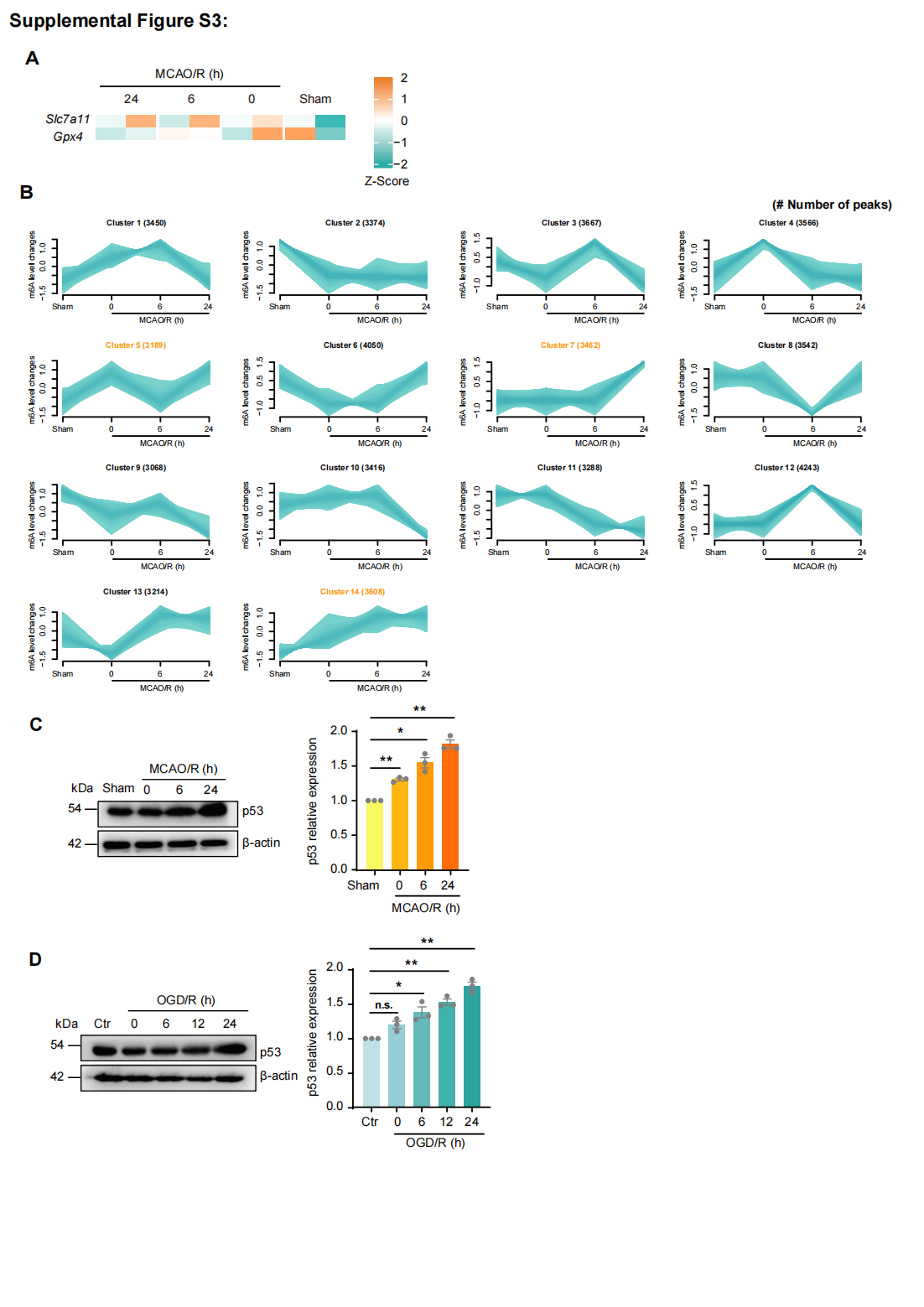


**Figure S3. Supplemental figure for Figure 3.** (A) Heat maps of changes in m^6^A modification levels for *Slc7a11* and *Gpx4* mRNA. (B) Fourteen clusters of genes were identified based on the temporal expression profile of their m^6^A methylation changes (see materials and methods). (C) Representative Western-blots and quantification of p53 protein in peri-infarct tissue post-MCAO/R. Data were expressed as means ± s.e.m. * *P*<0.05, ** *P*<0.01 versus Sham group. n=3 animals in each group. (D) Representative Western-blots and quantification of p53 protein in HT22 cells post-OGD/R. Data were expressed as means ± s.e.m. * *P*<0.05, ** *P*<0.01 versus Ctr group. n=3 experiments.

**Supplemental Figure S4:**


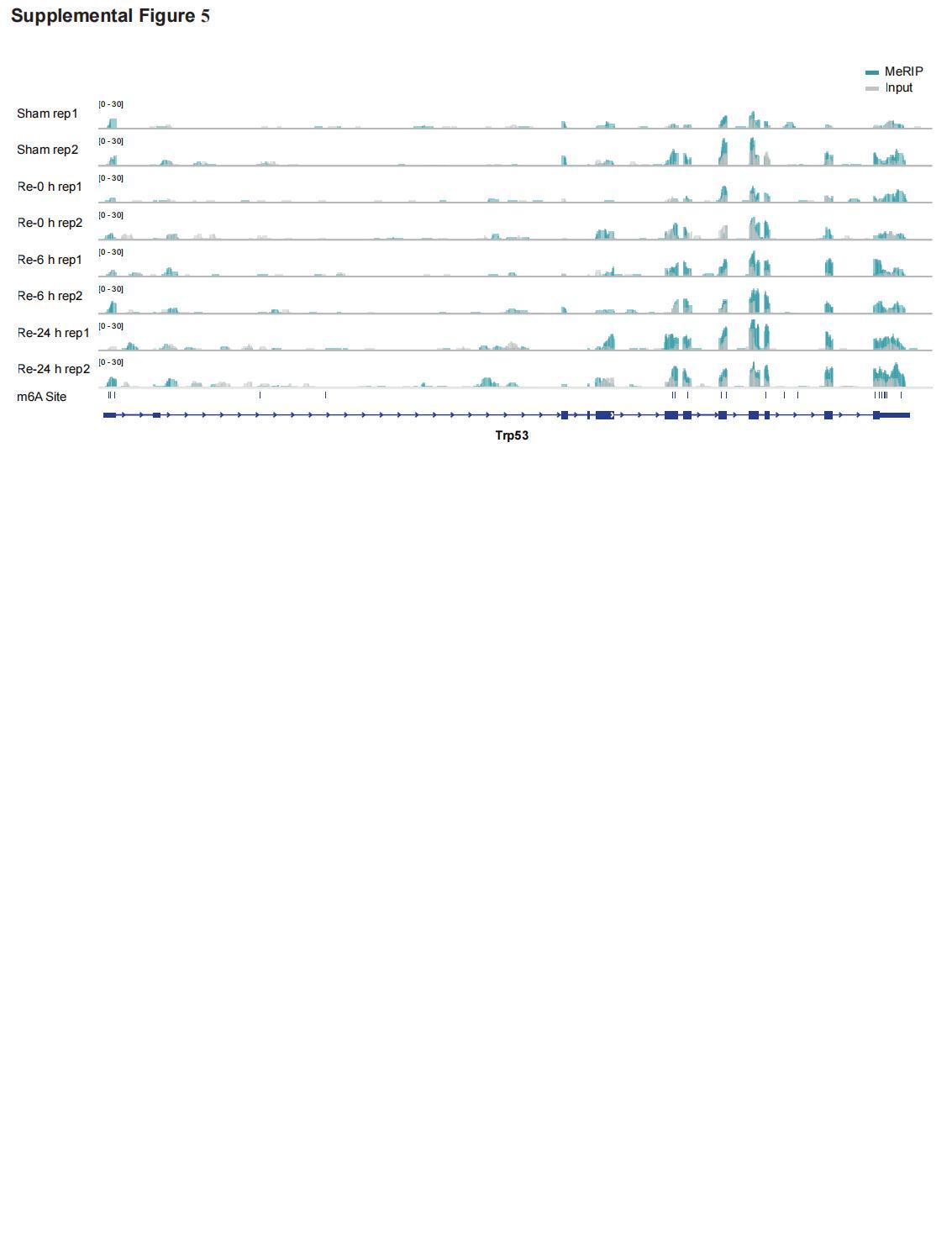


**Figure S4. Supplemental figure for Figure 3. Hypermethylated p53 mRNA after MCAO/R.** IGV view of MeRIP-seq read coverages of p53 (RNA input coverages in grey, m^6^A-IP coverages in blue) in Sham group and MCAO/R groups at different time points. n=2 experiments.

**Supplemental Figure S5:**


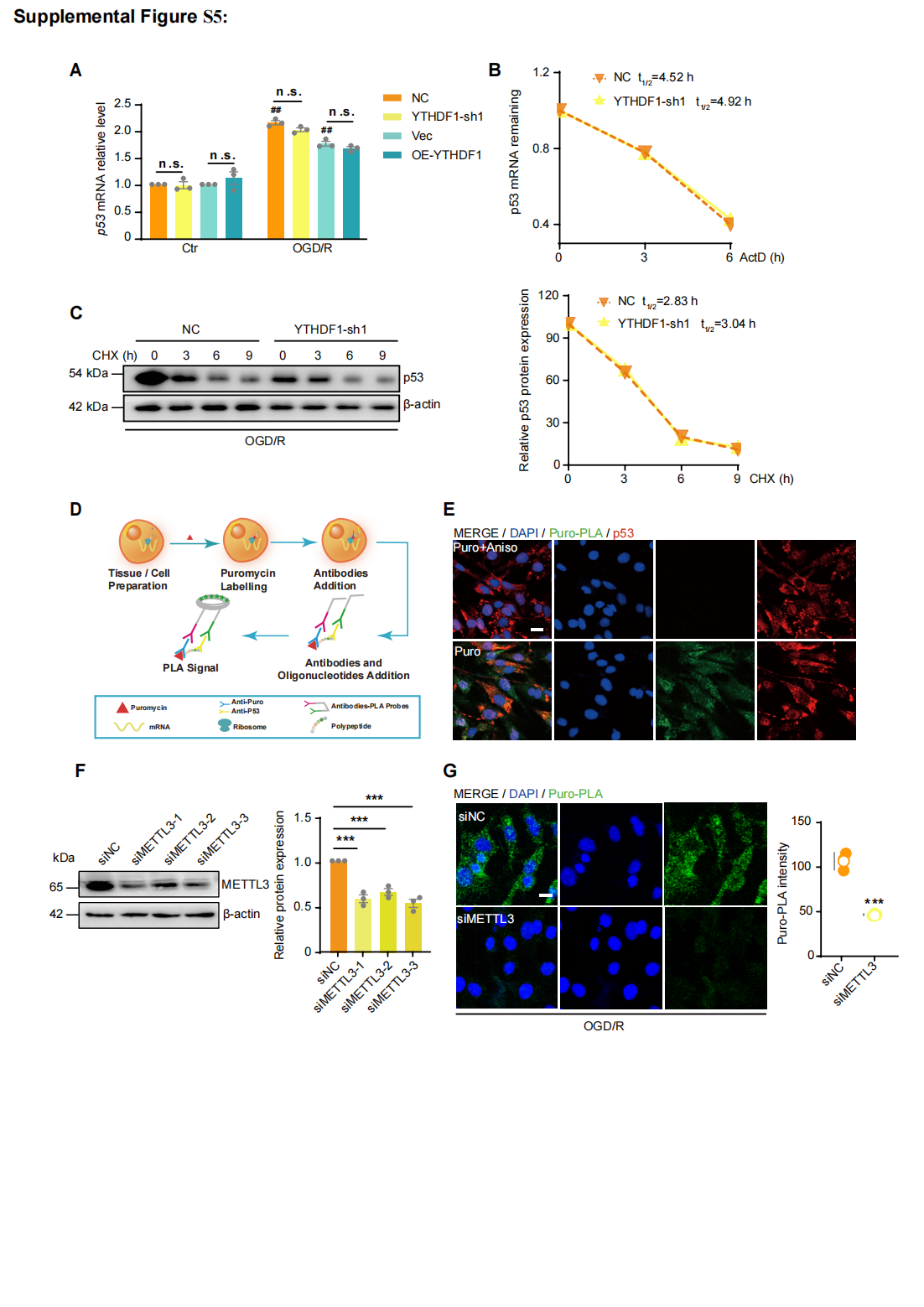


**Figure S5. Supplemental figure for Figure 4.** (A) *p53* mRNA levels in HT22 with YTHDF1 KD or OE with or without OGD/R. Data were expressed as means ± s.e.m. ##*P*<0.01 versus NC or Vec group. n=3 experiments. (B) *p53* mRNA degradation curve in HT22 cells with YTHDF1 KD and OGD/R. (C) Western-blots and quantification of p53 protein in HT22 cells with YTHDF1 KD and OGD/R. Data were expressed as means ± s.e.m. n=3 experiments. None of the above was affected by YTHDF1 KD or OE. (D) A schematic diagram of the Puro-PLA method (see Material and methods). (E) Confocal images of newly synthesized p53 protein with 10 min puromycin incubation (Puro-PLA signal in green, p53 immunostaining in red) in the presence (top row) or absence (bottom row) of the protein synthesis inhibitor Anisomycin (60 μM). Scale = 10 μm. Note that no Puro-PLA signal was observed in Anisomycin-treated cells. (F) Western-blot and quantification of METTL3 protein in HT22 transfected with siNC or siMETTL3-1/2/3. Data were expressed as means ± s.e.m. ****P*<0.001 versus siNC group. n=3. All three siMETTL3s successfully reduced METTL3 protein in HT22 cells. (G) Puro-PLA images detected the p53 translation in HT22 cells with siMETTL3 and OGD/R. Scale bar = 10 μm. Data were expressed as means ± s.e.m. ****P*<0.001 versus siNC group. n=3 experiments.

**Supplemental Figure S6:**


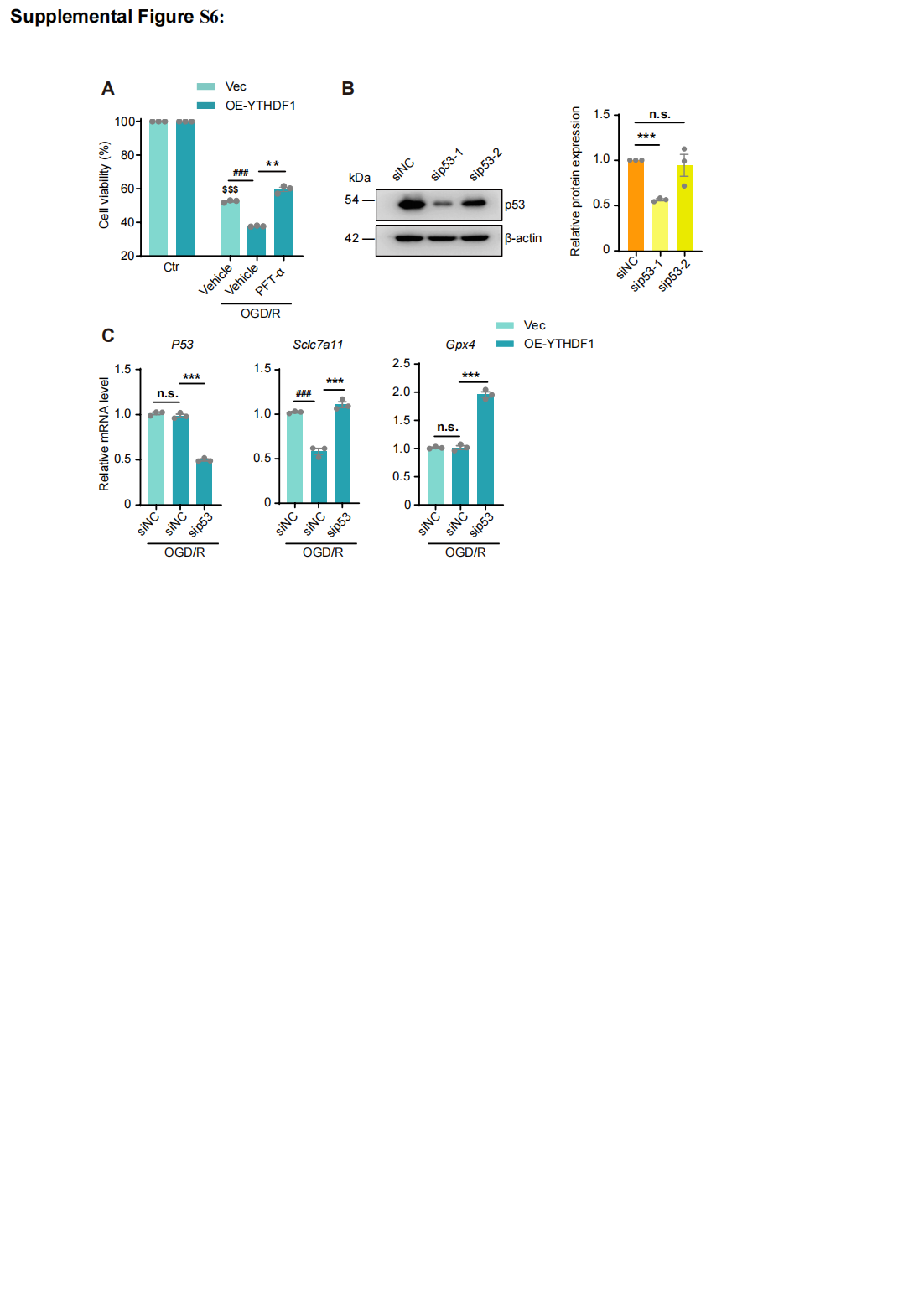


**Figure S6. Supplemental figure for Figure 5. Inhibiting p53 revoked YTHDF1 regulation of SLC7A11 and GPX4 expression post-OGD/R.** (A) Cell viability in YTHDF1-OE HT22 cells treated with Pifithrin-α (PFT-α, 2 μM; inhibits p53 transcription activity) during OGD/R. Data were expressed as means ± s.e.m. ***P* < 0.01 versus OGD/R and Vehicle group. ###*P*<0.001 versus Vec group. $$$*P*<0.001 versus Ctr group. n=3 experiments. (B) Western-blots and quantification of p53 in HT22 transfected with siNC or sip53-1/2. Data were expressed as means ± s.e.m. ****P*<0.001 versus siNC group. n=3 experiments. (C) Quantification of *p53*, *Slc7a11* and *Gpx4* mRNA in YTHDF1-OE HT22 cells post-OGD/R. Data were expressed as means ± s.e.m. ****P*<0.001 versus siNC group. ###*P*<0.001 versus Vec group, n=3 experiments.

**Supplemental Figure S7:**


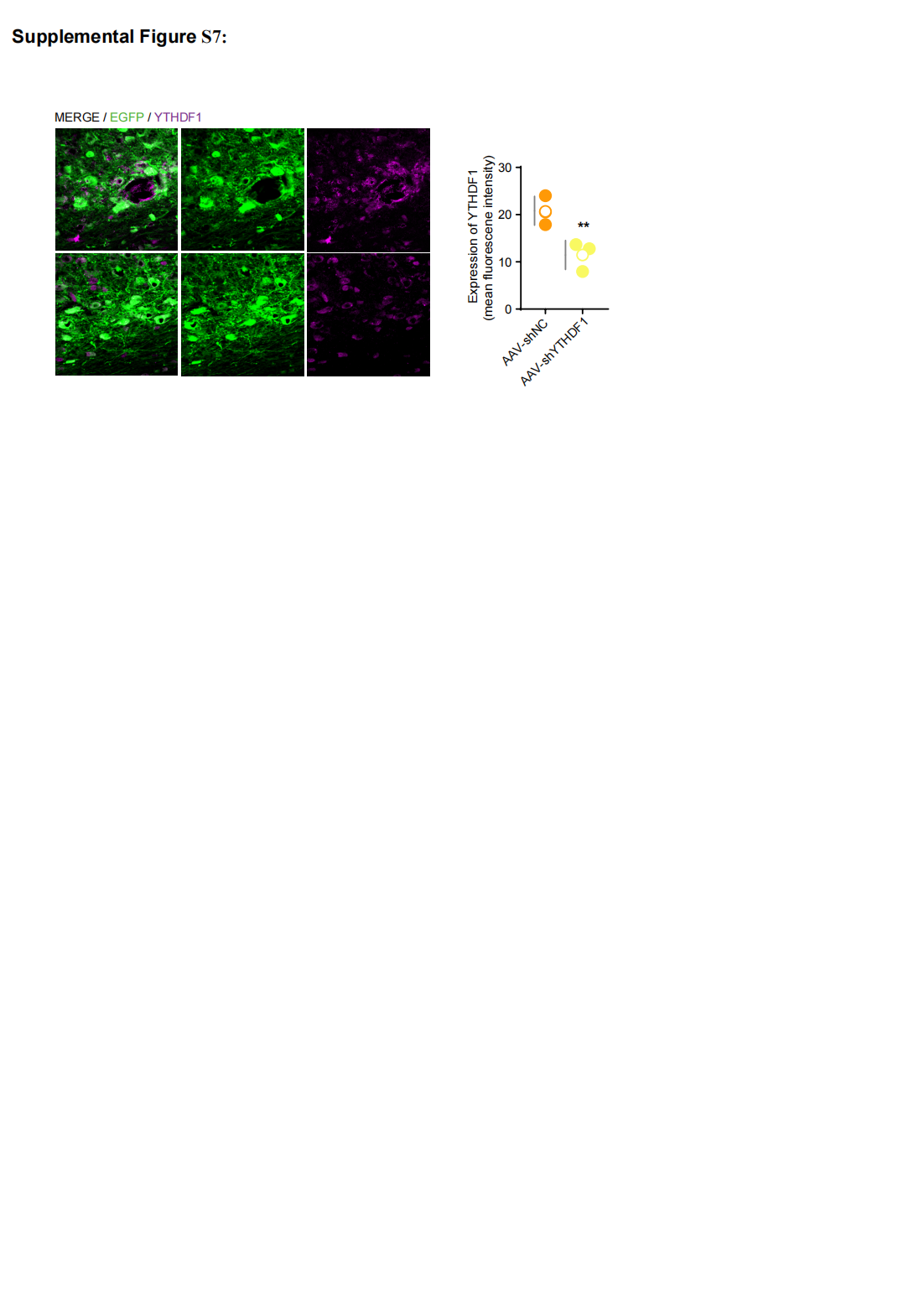


**Figure S7. Supplemental figure for Figure 6. Validation of neuronal YTHDF1 expression in AAV-shYTHDF1 infected cortical cortex tissue.** Immunofluorescence images of the mouse cerebral cortex 3 weeks after AAV-shYTHDF1 injection. YTHDF1 (Magenta) and EGFP (Green) were detected. Scale bar = 50 μm. Data were expressed as means ± s.e.m. ***P*<0.01 versus AAV-shNC group. n=3 animals per group.
